# The genus *Hebeloma* in the Rocky Mountain Alpine Zone

**DOI:** 10.3897/mycokeys.46.32823

**Published:** 2019-02-11

**Authors:** Cathy L. Cripps, Ursula Eberhardt, Nicole Schütz, Henry J. Beker, Vera S. Evenson, Egon Horak

**Affiliations:** 1 Plant Sciences and Plant Pathology, 119 Plant Biosciences Bldg, Montana State University, Bozeman, MT 59717, USA; 2 Staatliches Museum für Naturkunde Stuttgart, Rosenstein 1, D-70191 Stuttgart, Germany; 3 Rue Père de Deken 19, B-1040 Bruxelles, Belgium; Royal Holloway College, University of London, Egham, United Kingdom; Plantentuin Meise, Nieuwelaan 38, B-1860 Meise, Belgium; 4 Sam Mitchel Herbarium of Fungi, Denver Botanic Garden, 909 York Street, Denver, CO 80206, USA; 5 Institute of Microbiology, University of Innsbruck, Technikerstrasse 25, 6th floor, A-6020 Innsbruck, Austria

**Keywords:** A.H. Smith, Arctic-alpine, ectomycorrhizal, fungal biodiversity, *
Hymenogastraceae
*, ITS, systematics

## Abstract

Numerous taxa of *Hebeloma* have been reported in association with *Salix*, *Dryas*, and *Betula* in arctic-alpine habitats. However, species are notoriously difficult to delineate because morphological features overlap, and previously there was little reliable molecular data available. Recent progress in ITS-sequencing within the genus, coupled with an extensive database of parametrically described collections, now allows comparisons between species and their distributions. Here we report 16 species of *Hebeloma* from the Rocky Mountain alpine zone from some of the lowest latitudes (latitude 36°–45°N) and highest elevations (3000–4000 m) for arctic-alpine fungi in the northern hemisphere. Twelve of these species have been reported from arctic-alpine habitats in Europe and Greenland and are now molecularly confirmed from the Middle and Southern Rockies, greatly expanding their distribution. These are: *Hebelomaalpinum*, *H.aurantioumbrinum*, *H.dunense*, *H.hiemale*, *H.marginatulum*, *H.mesophaeum*, *H.nigellum*, *H.oreophilum*, *H.subconcolor*, *H.spetsbergense*, *H.vaccinum*, and *H.velutipes. Hebelomahygrophilum* is known from subalpine habitats in Europe, but was never recorded in arctic-alpine ecology. Three species recorded from the Rockies, but as yet not reported from Europe, are *H.alpinicola*, *H.avellaneum*, and *H.excedens*. The last two have never previously been reported from an arctic-alpine habitat. For all three of these species, the holotypes have been studied morphologically and molecularly, and have been incorporated into the analysis.

## Introduction

The alpine is defined as the life zone above treeline on high mountain tops and this biome constitutes 3% of the earth’s land (Körner 1999). In northern latitudes, it is characterized by low, open vegetation and a climate dominated by cold temperatures (Chapin and Körner 1995). Diurnal temperature fluctuations and periodic strong winds during the short growing season affect both plant development and basidiome production. Ectomycorrhizal fungi are critical to the survival of alpine woody plants such as *Salix*, *Dryas*, *Betula*, and non-woody plants such as Persicaria (Bistorta) and *Kobresia* in the alpine zone (Cripps and Eddington 2005). The most diverse ectomycorrhizal fungal genera in the Northern Hemisphere alpine are *Cortinarius*, *Inocybe*, *Hebeloma*, *Laccaria*, *Entoloma*, *Lactarius* and *Russula* (Gardes and Dahlberg 1996; Cripps and Horak 2008).

The Rocky Mountain alpine exists as islands on high mountain tops and plateaus separated by vast forests and grasslands. The middle and southern Rockies span some of the lowest latitudes (36°–45° N) and highest elevations (3000–4000 m) known for northern hemisphere alpine. Yet, species of *Inocybe* and *Lactarius* from the Rocky Mountain alpine zone have been found to be conspecific with those occurring in arctic and alpine habitats in the Alps, Pyrenees, Norway, Sweden, Finland, Svalbard, and Greenland through molecular matching of ITS (internally transcribed spacer) sequences (Cripps et al. 2010; Larsson et al. 2014; Barge et al. 2016; Barge and Cripps 2016).

The genus *Hebeloma* is common in arctic and alpine habitats, but species are poorly known. It is phylogenetically placed in the *Hymenogastraceae* Vittad. (Matheny et al. 2006) and is characterized by smooth to roughened brown spores that lack a visible germ pore, distinct cheilocystidia, an absence (usually) of pleurocystidia, and an ixocutis resulting in a smooth viscid pileus which is often two-colored (usually darker in the center). Distinctive odors, typically of radish or raw potato described as raphanoid are often present (Vesterholt 2005). However, not all species exhibit all features and character states overlap. Although most experienced mycologists will normally be able to identify a mushroom as a *Hebeloma* with relative ease, taxa are notoriously difficult to delineate at the species level because of variable morphological features and, until recently, a lack of reliable reference literature and a lack of confirmed DNA reference sequences of type materials. While the recent monograph by Beker et al. (2016) provides a great deal of reference material, this was centered on the *Hebeloma* of Europe; overlap between the European and American continents is currently being studied.

Numerous taxa of *Hebeloma* have been reported in association with *Persicaria*, *Betula*, *Salix*, and *Dryas* from arctic-alpine habitats including those in the Alps (Favre 1955; Bon 1986; Bruchet 1974; Debaud et al. 1981; Kühner and Lamoure 1986; Senn-Irlet 1990; Senn-Irlet 1993; Jamoni 2008; Graf 1994; Brunner et al. 2017), Iceland (Eyjolfsdottir 2009), Scandinavia (Vesterholt 2005, 2008; Knudsen and Vesterholt 2008), Svalbard (Hutinen 1987; Ohenoja 1971; Gulden and Torkelsen 1996; Beker et al. 2018), Pyrenees (Corriol 2008), and the Carpathians (Eberhardt et al. 2015b). In North America, there are reports from Greenland (Lange 1957; Borgen 2006;Borgen et al. 2006), Canada (Ohenoja and Ohenoja 1993, 2010), Alaska (Miller 1998), and the Rocky Mountains (Miller and Evenson 2001; Cripps and Horak 2008; Beker et al. 2010). A table comparing the occurrence of species in various arctic and alpine locations was presented in Beker et al. (2018); this table indicates 10 species from the Rocky Mountains. Beker and co-workers (2016) list 25 species occurring in arctic or alpine habitats, 14 of which appear (almost) restricted to these habitats; others also occur in a variety of habitats from subalpine or boreal with coniferous and hardwood trees right down to sand dunes where they grow with dwarf *Salix*. The veiled species of *Hebeloma* in Western North America have been treated in a monograph by Smith et al. (1983), but few (if any) of their collections are from above treeline, although many are from high elevations in the Rocky Mountains. While recent work on the genus *Hebeloma* in Europe now provides a basis for comparison of morphological and molecular data for a significant number of species and make possible comparisons of distribution patterns (Vesterholt 2005; Beker et al. 2016), much more work is needed before we will have a complete picture of the different species that occur on the different continents and their distribution across those continents. Here we delineate 16 species of veiled and unveiled *Hebeloma* primarily with *Salix* from the Rocky Mountain alpine zone. Thirteen of these taxa were described in detail in Beker et al. (2016) but three species described here were not included in that discussion of European *Hebeloma*. These three species (*H.alpinicola* A.H. Sm., Evenson & Mitchel, *H.avellaneum* Kauffman, and *H.excedens* (Peck) Sacc.), whose holotypes have been studied morphologically and molecularly, are described within this paper and their relationship with other *Hebeloma* species is explored.

As demonstrated in Beker et al. (2016), morphological differences do exist between species and although separation between species does need careful work, in almost all cases a morphological analysis may be used for determination of species and in some cases morphology is even better suited for species delimitation than the data of the five loci applied. Here we have carried out a morphological analysis to determine species and have found no conflict between our morphological placement and that provided by our molecular analysis based on ITS data. Tree and network building methods have been applied to demonstrate the taxonomic placement of the Rocky Mountains collections in relation to type specimens and confirmed collections of species treated by Beker et al. (2016). For the three species not treated in Beker et al. (2016) we include type sequences from American types. We do not provide lists of synonyms in the species descriptions, because we have not yet re-evaluated all species described outside Europe and any list that we could give would be provisional. Where we deem it necessary, synonyms are mentioned in species discussions. Species names and their synonyms from Europe have been treated to great detail by Beker et al. (2016).

A great majority of the encountered species was shown to be paraphyletic and part of species complexes by Beker et al. (2016) and previous works (Eberhardt et al. 2015a, 2016; Grilli et al. 2016). In the course of the studies for this work we found that the same is true for two species (*H.alpinicola* and *H.excedens*) not treated by Beker et al. (2016). We have chosen to illustrate the problems of species recognition and delimitation based on ITS data by showing networks for taxa treated by Eberhardt et al. (2015a, 2016) and Grilli et al. (2016), i.e. members of the *H.* sects. *Denudata* and *Velutipes*; and in addition to trees for members of H.sect.Hebeloma. The ITS region of members of these species complexes often differs only by a small number of base pairs between species, and comparable differences occur within species. Additionally, species often do not form monophyla within these complexes.

Median-Joining Networks have been recommended for inferring intraspecific phylogenies (i.e. Bandelt et al. 1999). Pruned quasi-median networks (Ayling and Brown 2008) are a tool to visualize DNA sequence variation when evolution has not necessarily been treelike. No assumptions are made as to which evolutionary mechanisms (i.e. hybridization, recombination, etc.) have been responsible for the observed variation. In the networks, observed sequence variants are shown as circles and the size of each circle represents the number of times the respective sequence variant has been observed. Two circles connected by an unsegmented line differ in 1 bp. So-called quasi-medians, a kind of placeholder for unobserved sequence variants, are placed between observed sequence variants that each differ from the quasi-median by 1 bp. The number of segments to a line represents the number of base pair changes between two sequence variants or a sequence variant and a quasi-median. A pruning mechanism is applied to reduce the complexity of the networks while depicting at least one shortest path between all pairs of sequence variants (Ayling and Brown 2008).

Ideally, we would have been able to present networks of haplotypes. What we here refer to as ‘ITS variants’ are sequencing results of dikaryotic material; in many cases, the sequences do not seem to correspond to a single haplotype. Although the ITS exists in multiple copies in the genome, it has been shown to behave like a dikaryotic locus in *Hebeloma* (Aanen et al. 2001) and other fungi (i.e. Schnabel et al. 2005; Hughes et al. 2013). Even good quality reads of ITS and other nuclear loci of many *Hebeloma* species contain one or several ambiguous positions and/or indications of indels, which we consider as evidence of variation between haplotypes of the same locus. Here, the level of variation was such that attempts to phase all ITS data into haplotypes (Flot et al. 2006; Flot 2010) were aborted and each collection is represented by a single ITS variant, i.e. the consensus sequence of both ‘haplotypes’.

## Methods

### Study sites

**Figure 1.**
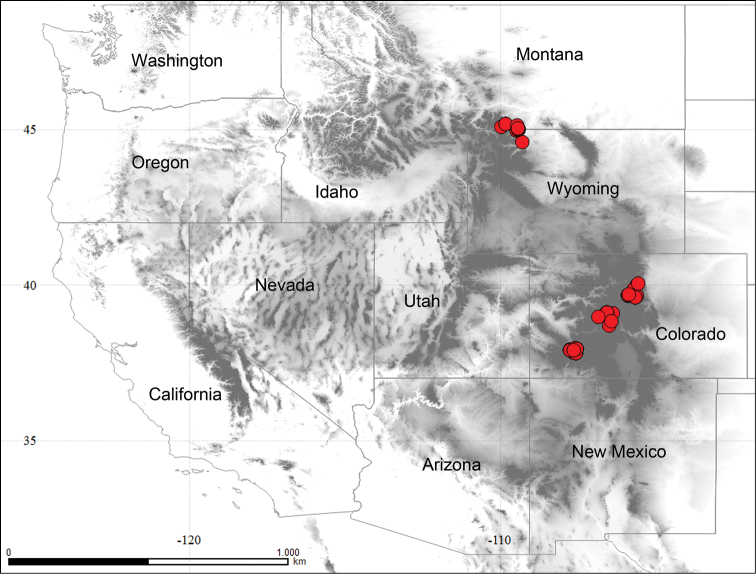
Distribution of Rocky Mountain alpine collections of *Hebeloma*. The map was generated with QGIS version 2.2.0 using WGS84 (EPDG: 4326; QGIS Development Team 2018). Shapefiles were provided by the Database of Global Administrative Areas (GADM, https://gadm.org/), accessed April 2018.

Our primary study sites are in the Middle-Northern and Southern Floristic zones of the Rocky Mountains that extend from Montana to Colorado (Fig. 1); the phytogeography is described in Cripps and Horak (2008) and further site details are in Barge et al. (2016) and Osmundson et al. (2005). Primary collecting sites include the Beartooth Plateau (latitude 45° N, elevation 3000–3500 m) in Montana and Wyoming, and the Front Range, Sawatch Range, and San Juan Mountains in Colorado (latitude 36°–38° N, elevation 3600–4000 m). Ectomycorrhizal vascular plants include *Salixreticulata*, *S.arctica*, *S.rotundifolia*, *S.cascadensis*, *S.planifolia*, *S.glauca*, *Betulaglandulosa* (= *B.nana*), *Dryasoctopetala*, *Persicariavivipara*, and *Kobresiamysuroides* (Cripps and Eddington 2005). While our study was focused on areas of tundra above the tree line, occasionally small *Picea* shrubs also occurred and it was not possible to unambiguously specify the mycorrhizal partner.

### Collections and morphological descriptions

Basidiomes were collected from late July through August, which constitutes the field season, from 1980 to 2017. Most collections were described in fresh condition, photographed, and dried on a dehydrator overnight. Dehydrated material was deposited in the MONT herbarium (Montana State University), ETH (Zurich, Switzerland), DBG (Denver Botanic Gardens), and/or the HJB private herbarium. Microscopic examination of dried material was done in 5% KOH to measure spores, cystidia, basidia, and other important features and in Melzer’s solution to assess dextrinoid reactions following Beker et al. (2016) and Vesterholt (2005). Within the species descriptions below we conform to spore descriptions based on spore ornamentation measures (O1–O4), spore dextrinoidity measures (D0–D3) and perispore loosening measure (P0–P3), as described in Beker et al. (2016). Similarly, cheilocystidia measurements include length, maximum width near the apex, minimum width in the median part of the cystidium and maximum width in the basal part of the cystidium. No distinction is made in the spore measurements for spores from two- and four-spored basidia. Measurements for the two types of spores are given separately in the Suppl. material 1. Exsiccate were also described. Unless otherwise mentioned, the species descriptions given are based on the collections from the Rocky Mountains cited here.

### Molecular analyses

ITS sequence data from the 115 *Hebeloma* collections from the Rocky Mountains (which is referred to as the RM dataset), 221 reference sequences including some type sequences from Europe (referred to as the FE (Fungi Europaei) dataset, see Beker et al. 2016) and 10 type collections of species described from the US, pertinent to the RM collections, were generated using a variety of protocols (Eberhardt 2012; Eberhardt et al. 2016). Newly generated sequences were submitted to GenBank (acc. no. MK280985–MK281025, MK286558–MK286561, and MK305906–MK305939).

The DNA of old material was extracted using the Gentra Puregene kit (Qiagen, Hilden, Germany), modifying the procedure that is described in the manual (version 2014) for yeasts, generally replacing any pipetting of DNA-containing fluids by pouring (see Eberhardt et al. 2016). A small amount of basidiome material was crushed in a TissueLyser II (Qiagen), suspended in 300 µl suspension solution plus 1.5 µl lytic enzyme for 30 min at 37 °C. The samples were centrifuged for 5 min at 8000 rpm and the supernatant poured out. Lysis was done in 300 µl of Cell Lysis Solution, the samples mixed by vortexing and incubated overnight at 37 °C, followed by 1 h at 65 °C. Samples were cooled to room temperature and 100 µl Protein Precipitation Solution added. Prior to centrifugation (maximum speed, 5 min), the samples were placed in the freezer for 10–15 min. Each sample was then poured into a prepared tube with 300 µl absolute isopropanol and 1 µl of glycogen (Life Technologies, Darmstadt; diluted 1:1 with ultrapure water). After mixing by repeatedly inverting for 1 min, the DNA was precipitated overnight to several days in the fridge. The pellets were washed in 300 µl 70% ethanol, air-dried for 30 min and re-desolved in 50 µl DNA Hydration Solution. The purified DNA was re-desolved by heating the samples for one hour at 65 °C and keeping them overnight at room temperature. DNA extracts were diluted for PCR as required. ITS1 and ITS2 were amplified separately in 35–40 cycles of PCR (30 s denaturation at 95 °C, 45 s annealing at 55 °C, and 60 s elongation at 72 °C) with 1.25 U/25 µl MyTaq Red (Bioline, Luckenwalde, Germany), using the primer pairs ITS1F/ITS2 and 58SF/ITS4 (White et al. 1990; Gardes and Bruns 1993; Tedersoo et al. 2013 [who erroneously ascribed the primer 58SF (3' - ATG CAT CGA TGA AGA ACG C -5' to Martin and Rygiewicz 2005]). Sequencing was carried out at LGC (Berlin, Germany).

Taxonomic assignment to section and species cluster was done via BLAST searches against the collections analyzed in depth by Beker et al. (2016), the FE dataset, in Geneious R10 (version 10.2.3, Biolmatters, Auckland, NZ). To illustrate the taxonomic placement of the RM collections, eight alignments were assembled using Mafft online with the G-INS-I option (Katoh et al. 2017), breaking up the large number of sequences into manageable datasets based on BLAST results. Alignments include RM and FE representatives of the target species, i.e. species occurring in the Rockies, relevant types for non-European species, and (where applicable) FE sequences of taxa that cannot be unambiguously distinguished from the target taxa, i.e. neither target species nor sister species forming monophyla in the ITS analyses of Beker et al. (2016) for arctic-alpine species. For better readability, non-arctic-alpine sister species clearly distinct from the target species were excluded from the final analyses. Species excluded from the analyses were *H.crustuliniforme* (Bull.) Quél. and *H.salicicola* Beker, Vesterh. & U. Eberh. for the *H.alpinum* complex; *H.psammophilum* Bon and *H.subtortum* P. Karst for the *H.mesophaeum* complex; as well as *H.monticola* Vesterh. and *H.fuscatum* for the *H.nigellum* complex. Also, for better readability, the number of European representatives of the included species was restricted to 10 (if available) or, for species present in the RM dataset in more than 10 collections, matching (if possible) the number of collections of the RM dataset. An exception was made for *H.velutipes*, for which 20 sequences were included because of the known high intraspecific diversity of this species. For each included species, the selection of included representatives from Beker et al. (2016) was random, but only considering sequences with high quality reads. For illustrating the placement of *H.avellaneum*, not included in Beker et al. (2016), a small alignment was assembled representing all species accepted by Beker et al. (2016) in H.sect.Naviculospora. For tree analyses, outgroup sequences were added; selection of outgroup taxa followed Beker et al. (2016). Details are given in Table 1 for the sequences of Rockies collections, in Table 2 for other American collections, the majority types, and in Suppl. material 1 for FE data (Supplementary Data). Alignments were viewed and reformatted using AliView version 1.24 (Larsson 2014) and have been submitted to TreeBase (http://purl.org/phylo/treebase/phylows/study/TB2:S23704). In summary, seven networks were calculated, one each for *H.alpinum* (J. Favre) Bruchet, *H.aurantioumbrinum* Beker, Vesterh. & U. Eberh., *H.hiemale* Bres. and *H.vaccinum* Romagn. *Hebelomasubconcolor* Bruchet and *H.velutipes* Bruchet are treated together, as are *H.excedens*, *H.marginatulum* (J. Favre) Bruchet, *H.mesophaeum* (Pers.) Quèl., and *H.alpinicola* as well as *H.hygrophilum* Poumarat & Corriol, *H.nigellum* Bruchet, *H.spetsbergense* Beker & U. Eberh., and *H.oreophilum* Beker & U. Eberh.

**Table 1.** Taxon, voucher (Herbarium), locality information, elevation, and GenBank accession numbers for DNA sequences from Rockies collections described here. HJB refers to the herbarium of H.J. Beker; other herbarium acronyms follow Thiers http://sweetgum.nybg.org/ih/(continuously updated). The database numbers refer to the project database of H.J. Beker (Beker et al. 2016).

Database no.	Herbarium	Voucher	Location	State	Elev. (m)	GenBank acc. no. ITS
*** Hebeloma alpinum ***						
HJB15331	MONT; HJB	CLC2855	Lulu Pass, near Cooke City	USA: MT	3000	MK281073
*** Hebeloma aurantioumbrinum ***						
HJB12445	HJB	HJB12445	Beartooth Plateau, Wyoming Creek	USA: WY	3176	KM390714, KM390715
HJB12446	HJB	HJB12446	Beartooth Plateau, Wyoming Creek	USA: WY	3176	KM390716, KM390717
HJB12447	HJB	HJB12447	Beartooth Plateau, Wyoming Creek	USA: WY	3176	MK281061
HJB12448	HJB	HJB12448	Beartooth Plateau, Wyoming Creek	USA: WY	3177	KM390718, KM390719
HJB12450	HJB	HJB12450	Beartooth Plateau, Wyoming Creek	USA: WY	3177	MK281062
HJB12451	HJB	HJB12451	Beartooth Plateau, Wyoming Creek	USA: WY	3177	KM390720, KM390721
HJB12452	HJB	HJB12452	Beartooth Plateau, Wyoming Creek	USA: WY	3177	MK281059
HJB12453	HJB	HJB12453	Beartooth Plateau, Wyoming Creek	USA: WY	3177	MK281063
HJB12454	HJB	HJB12454	Beartooth Plateau, Wyoming Creek	USA: WY	3177	MK281060
HJB12456	HJB	HJB12456	Beartooth Plateau, Wyoming Creek	USA: WY	3176	KM390722
HJB12583	ZT; HJB	ZT12730	Beartooth Mts., Hellroaring Plateau	USA: MT	3400	MK281119
HJB12584	ZT; HJB	ZT12731	Beartooth Mts., Hellroaring Plateau	USA: MT	3400	MK281118
HJB15300	MONT; HJB	CLC1565	Beartooth Plateau, Highline Trail	USA: MT	3100	MK281076
HJB15316	MONT; HJB	CLC1822	San Juan Range, Stony Pass	USA: CO	3840	MK281074
HJB15332	MONT; HJB	CLC3093	Beartooth Plateau, Frozen Lake	USA: WY	3200	MK281075
*** Hebeloma avellaneum ***						
HJB15496	DBG	DBG-F-020434	Front Range, Loveland Pass Lake	USA: CO	3620	MK281025
HJB15525	DBG	DBG-F-019533	Front Range, Niwott Ridge	USA: CO	3200	MK281026
*** Hebeloma dunense ***						
HJB12578	ZT; HJB	ZT9001	San Juan Range, Cinnamon Pass W	USA: CO	3700	MK281120
HJB15290	MONT; HJB	CLC1411	San Juan Range, Cinnamon Pass	USA: CO	3700	MK281079
HJB15293	MONT; HJB	CLC1434	San Juan Range, Cinnamon Pass	USA: CO	3700	MK281080
HJB15315	MONT; HJB	CLC1821	San Juan Range, Stony Pass	USA: CO	3840	MK281077
HJB15321	MONT; HJB	CLC1845	San Juan Range, Mineral Basin	USA: CO	3835	MK281078
*** Hebeloma excedens ***						
HJB12573	ZT; HJB	ZT7475	Sawatch Range, Independence Pass	USA: CO	3760	MK281122
HJB12575	ZT; HJB	ZT8074	Front Range, Loveland Pass	USA: CO	3750	MK281124
HJB12577	ZT; HJB	ZT8136	Sawatch Range, Independence Pass	USA: CO	3680	MK281123
HJB12582	ZT; HJB	ZT9830	Sawatch Range, Independence Pass	USA: CO	3700	MK281121
HJB15308	MONT; HJB	CLC1685	San Juan Range, U.S. Basin	USA: CO	3658	MK281081
HJB15312	MONT; HJB	CLC1732	Sawatch Range, Independence Pass	USA: CO	3760	MK281082
*** Hebeloma hiemale ***						
HJB12457	HJB	HJB12457	Beartooth Plateau, Quad Creek	USA: MT	3004	GQ869529
HJB12571	ZT; HJB	ZT6417	Beartooth Plateau, Highline Trail	USA: WY	3200	GQ869530
HJB12574	ZT; HJB	ZT8072	Front Range, Loveland Pass	USA: CO	3750	MK281083
HJB12581	ZT; HJB	ZT9828	Sawatch Range, Independence Pass	USA: CO	3750	MK281084
HJB15301	MONT; HJB	CLC1574	Beartooth Plateau, Quad Creek	USA: MT	3020	MK281037
HJB15306	MONT; HJB	CLC1668	San Juan Range, Mineral Basin,	USA: CO	3835	MK281027
HJB15333	MONT; HJB	CLC3094	Beartooth Plateau, Frozen Lake	USA: WY	3200	MK281028
HJB15493	DBG	DBG-F-019162	Front Range, Loveland Pass	USA: CO	3655	MK281029
HJB15495	DBG	DBG-F-021418	Front Range, Loveland Pass	USA: CO	3620	MK281030
HJB15497	DBG	DBG-F-020440	Front Range, Loveland Pass	USA: CO	3597	MK281031
HJB15498	DBG	DBG-F-020437	Front Range, Loveland Pass	USA: CO	3655	MK281032
HJB15499	DBG	DBG-F-019241	Front Range, Loveland Pass	USA: CO	3749	MK281033
HJB15500	DBG	DBG-F-020551	Front Range, Mt. Goliath	USA: CO	3658	MK281038
HJB15501	DBG	DBG-F-021194	Front Range, Loveland Pass	USA: CO	3620	MK281036
HJB15502	DBG	DBG-F-020431	Front Range, Loveland Pass	USA: CO	3597	MK281034
HJB15503	DBG	DBG-F-020433	Front Range, Loveland Pass	USA: CO	3571	MK281035
HJB15518	DBG	DBG-F-019597	Front Range, Loveland Pass	USA: CO	3620	MK281067
HJB15519	DBG	DBG-F-016104	Front Range, W Caribou townsite	USA: CO	3200	MK281068
HJB15520	DBG	DBG-F-020550	Front Range, Mt. Goliath	USA: CO	3810	MK281069
HJB17303	MONT; HJB	CLC3574	Beartooth Plateau, site 1	USA: MT	3000	GQ869526
HJB17304	MONT; HJB	CLC3575	Beartooth Plateau, site 1	USA: MT	3000	GQ869528
HJB17307	MONT; HJB	CLC3533	Beartooth Plateau, site 1	USA: MT	3000	MK281085
*** Hebeloma hygrophilum ***						
HJB15296	MONT; HJB	CLC1462	Sawatch Range, Independence Pass	USA: CO	3760	MK281086
HJB15297	MONT; HJB	CLC1476	Sawatch Range, Independence Pass	USA: CO	3660	MK281088
HJB15329	MONT; HJB	CLC1948	Beartooth Plateau, Frozen Lake	USA: MT	3200	MK281087
HJB15531	DBG	DBG-F-021349	Front Range, Loveland Pass	USA: CO	3658	MK281039
*** Hebeloma marginatulum ***						
HJB12458	HJB	HJB12458	Beartooth Plateau, Quad Creek	USA: MT	2996	MK281064
HJB12579	ZT; HJB	ZT9002	San Juan Range, Cinnamon Pass	USA: CO	3800	MK281126
HJB12580	ZT; HJB	ZT9813	San Juan Range, Black Bear Pass	USA: CO	3900	MK281125
HJB15291	MONT; HJB	CLC1413	San Juan Range, Cinnamon Pass,	USA: CO	3700	MK281089
HJB15294	MONT; HJB	CLC1448	San Juan Range, Black Bear Basin	USA: CO	3830	MK281090
HJB15295	MONT; HJB	CLC1449	San Juan Range, Black Bear Basin	USA: CO	3830	MK281091
HJB15298	MONT; HJB	CLC1478	Sawatch Range. Independence Pass	USA: CO	3760	MK281100
HJB15299	MONT; HJB	CLC1545	Beartooth Plateau, Quad Creek	USA: MT	3020	MK281092
HJB15305	MONT; HJB	CLC1667	San Juan Range, Mineral Basin	USA: CO	3835	MK281093
HJB15310	MONT; HJB	CLC1718	San Juan Range, Black Bear Basin	USA: CO	3760	MK281103
HJB15314	MONT; HJB	CLC1811	San Juan Range, Cinnamon Pass	USA: CO	3700	MK281094
HJB15317	MONT; HJB	CLC1824	San Juan Range, Stony Pass	USA: CO	3840	MK281095
HJB15318	MONT; HJB	CLC1826	San Juan Range, Stony Pass	USA: CO	3840	MK281101
HJB15319	MONT; HJB	CLC1836	San Juan Range, Imogene Pass	USA: CO	3850	MK281102
HJB15320	MONT; HJB	CLC1840	San Juan Range, Imogene Pass	USA: CO	3850	MK281096
HJB15322	MONT; HJB	CLC1860	San Juan Range, Mineral Basin	USA: CO	3835	MK281097
HJB15323	MONT; HJB	CLC1861	Mineral Basin, San Juan Range	USA: CO	3835	MK281104
HJB15324	MONT; HJB	CLC1874	San Juan Range, Emma Lake	USA: CO	3688	MK281098
HJB15326	MONT; HJB	CLC1880	San Juan Range, Emma Lake	USA: CO	3688	MK281099
HJB15487	DBG	DBG-F-027694	Front Range, Loveland Pass	USA: CO	3911	MK281048
HJB15488	DBG	DBG-F-027695	Front Range, Summit Lake Park	USA: CO	3911	MK281040
HJB15491	DBG	DBG-F-027682	Front Range, Summit Lake Park	USA: CO	3911	MK281041
HJB15505	DBG	DBG-F-020708	Front Range, Loveland Pass	USA: CO	3655	MK281042
HJB15506	DBG	DBG-F-020841	Sawatch Range, Independence Pass	USA: CO	3687	MK281046
HJB15507	DBG	DBG-F-020856	Sawatch Range, Independence Pass	USA: CO	3687	MK281047
HJB15512	DBG	DBG-F-021405	Front Range, Loveland Pass	USA: CO	3620	MK281043
HJB15533	DBG	DBG-F-021388	Front Range, Loveland Pass	USA: CO	3655	MK281044
HJB15534	DBG	DBG-F-020843	Sawatch Range, Independence Pass	USA: CO	3687	MK281045
HJB17308	MONT; HJB	CLC3545	Beartooth Plateau, Solufluction Terr	USA: WY	3400	MK281070
*** Hebeloma mesophaeum ***						
HJB12576	ZT; HJB	ZT8082	Front Range, Loveland Pass	USA: CO	3750	MK281127
HJB15289	MONT; HJB	CLC1245	Sawatch Range, Independence Pass	USA: CO	3760	MK281105
*** Hebeloma nigellum ***						
HJB12572	ZT; HJB	ZT6425	Beartooth Plateau, Pass N	USA: WY	3350	MK281128
HJB15292	MONT; HJB	CLC1420	San Juan Range, Engineer Pass	USA: CO	3900	MK281106
HJB15309	MONT; HJB	CLC1707	San Juan Range, Cinnamon Pass	USA: CO	3700	MK281107
HJB15313	MONT; HJB	CLC1778	Beartooth Plateau, Frozen Lake	USA: WY	3200	MK281108
HJB17305	MONT; HJB	CLC3614b	Beartooth Plateau, Billings Fen	USA: WY	3400	MK281071
*** Hebeloma nigromaculatum ***						
HJB12439	HJB	HJB12439	Beartooth Plateau, Quad Creek	USA: MT	2988	MK281065
HJB15302	MONT; HJB	CLC1577	Beartooth Plateau, Quad Creek	USA: MT	3020	MK281109
HJB15529	DBG	DBG-F-020565	Front Range, Little Echo Lake	USA: CO	3505	MK281050
HJB15530	DBG	DBG-F-020582	Front Range, Little Echo Lake	USA: CO	3505	MK281049
*** Hebeloma oreophilum ***						
HJB12449	HJB	HJB12449	Beartooth Plateau, Wyoming Creek	USA: WY	3176	MK281066
HJB12585	ZT; HJB	ZT12733	Beartooth Mts., Hellroaring Plateau	USA: MT	3400	MK281129
HJB15288	MONT; HJB	CLC1102	Beartooth Plateau, Quad Creek	USA: MT	3020	MK281110
HJB15328	MONT; HJB	CLC1937	Beartooth Plateau, Highline Trail	USA: MT	3100	MK281111
HJB15489	DBG	DBG-F-027674	Front Range, Summit Lake Park	USA: CO	3911	MK281054
HJB15504	DBG	DBG-F-022788	Front Range, Summit Lake Park	USA: CO	3912	MK281051
HJB15508	DBG	DBG-F-020053	Elk Mountain Range, Pearl Pass	USA: CO	3658	MK281052
HJB15521	DBG	DBG-F-020558	Front Range, Mount Goliath	USA: CO	3658	MK281053
HJB17306	MONT; HJB	CLC3607	Beartooth Plateau, Billings Fen	USA: WY	3048	MK281072
*** Hebeloma spetsbergense ***						
HJB15325	MONT; HJB	CLC1879	San Juan Range, Horseshoe Basin	USA: CO	3688	MK281112
HJB15490	DBG	DBG-F-027678	Front Range, Summit Lake Park	USA: CO	3911	MK281055
*** Hebeloma subconcolor ***						
HJB15510	DBG	DBG-F-022785	Front Range, Summit Lake Park	USA: CO	3912	MK281056
HJB15511	DBG	DBG-F-022786	Front Range, Summit Lake Park	USA: CO	3912	MK281057
*** Hebeloma vaccinum ***						
HJB15327	MONT; HJB	CLC1881	San Juan Range, Horseshoe Basin	USA: CO	3688	MK281113
*** Hebeloma velutipes ***						
HJB12570	ZT; HJB	ZT6100	Beartooth Plateau, N of E Summit	USA: MT	3320	MK281130
HJB15303	MONT; HJB	CLC1646	Sawatch Range, Cottonwood Pass	USA: CO	3694	MK281116
HJB15304	MONT; HJB	CLC1651	Sawatch Range, Cumberland Pass	USA: CO	3668	MK281117
HJB15311	MONT; HJB	CLC1725	Sawatch Range, Cottonwood Pass	USA: CO	3694	MK281115
HJB15330	MONT; HJB	CLC1980	Beartooth Plateau, Quad Creek	USA: MT	3020	MK281114
HJB15524	DBG	DBG-F-005617	Front Range, Herman Gulch	USA: CO	3170	MK281058

**Table 2.** Other North American collections considered. HJB refers to the herbarium of H.J. Beker; other herbarium acronyms follow Thiers http://sweetgum.nybg.org/ih/(continuously updated). The database numbers refer to the project database of H.J. Beker (Beker et al. 2016).

Database no.	Herbarium	Voucher	Location	State	Elev. (m)	GenBank acc. no. ITS
*** Hebeloma alpinicola ***						
HJB1000311	MICH	MICH 5549†	Heavens Gate Ridge, Seven Devils Mountains	USA: Idaho	2560	MK280987
HJB1000338	DBG	DBG-F-002473‡	Park County, Pike National Forest, Sacramento, west of Fairplay, north side of old house	USA: Colorado	3600	MK286559
HJB1000416	MICH	MICH 10760§	Hancock, Bar Harbor, Mt Desert Island	USA: Maine	25	MK286558
HJB1000435	MICH	MICH 10778|	Clackamas, Rhododendron	USA: Oregon	495	MK280989
HJB1000500	DBG	DBG-F-004877¶	Gilpin County, Roosevelt National Forest, Perigo, north slope	USA: Colorado	2865	MK286560
HJB1000147	MICH	MICH 10730#	Chelsea, Lyndon Town Hall Park, Washtenaw Co.	USA: Michigan	300	MK280985
HJB1000501	DBG	DBG-F-007947††	Conejos County, San Juan National Forest, Green Lake area south of Platero	USA: Colorado	3353	MK286561
*** Hebeloma avellaneum ***						
HJB14320	FNL‡‡; HJB	HJB14320	Pinware River	Canada: Labrador	15	MK281019
HJB1000322	MICH§§	MICH 10722	Grays Harbor, Lake Quinault, Olympic National Park	USA: Washington	75	MK280988
*** Hebeloma excedens ***						
HJB1000268	NYS	NYS-F-001123||	Saratoga, Saratoga	USA: New York	100	MK280986
*** Hebeloma incarnatulum ***						
HJB1000136	MICH	MICH 10752¶¶	Mud Lake Bog west of Whitmore Lake, Washtenaw	USA: Michigan	275	KT218477

†This is the holotype of *Hebelomaalpinicola*, 5 Jul 1958, A.H. Smith (58632). ‡This is the holotype of *Hebelomachapmaniae*, 10 Sep 1969, S. Chapman. §This is the holotype of *Hebelomalittenii*, 29 Oct 1980, W. Litten. |This is the holotype of *Hebelomanigromaculatum*, 1 Oct 1944, A.H. Smith (19314). ¶This is the holotype of *Hebelomaperigoense*, 13 Aug 1974, S. Chapman, S. Mitchel, A.H. Smith. #This is the holotype of *Hebelomasmithii*, 10 Nov 1977, A.H. Smith (88295). ††This is the holotype of *Hebelomasubargillaceum*, 23 Aug 1978, V. Evenson. ‡‡Foray Newfoundland and Labrador herbarium http://www.nlmushrooms.ca/index.html §§This is the holotype of *Hebelomaavellaneum*, 8 Nov 1925, C.H. Kauffman. ||This is the holotype of *Hebelomaexcedens*, Oct 1870, C.H. Peck. ¶¶This is the holotype of *Hebelomaincarnatulum*, 14 Oct 1961, A.H. Smith (64680).

Maximum Likelihood analyses were calculated in RaxML (version 8.2.10, Stamatakis 2014) as implemented on Cipres (Miller et al. 2010), with the GTRGAMMA option, five searches for the best ML tree, using the MRE option to limit the number of fast bootstrap replicates. Trees were visualized using FigTree version 1.4.2 (Rambaut 2014).

Pruned quasi-median network analyses were carried out in SplitsTree (version 4.14.6, Huson and Bryant 2006) using the default settings apart from activating the ‘scale nodes by taxa’ and ‘subdivide edges’ options. Nodes representing different classes of sequences (differentiated by species and origin, RM versus FE) were replaced in Adobe Illustrator CS6 by pie charts of corresponding diameters, showing the relative numbers of sequences for each class.

Distances between sequences were calculated in PAUP* (Swofford 2003), as the total number of differences of standard data, disabling the default ‘equate’ scheme for sequence data. By doing this, ambiguity reads like i.e. ‘y’ are not equated with the corresponding bases, here ‘c’ and ‘t’. Missing data were recoded as ‘?’; gaps were treated as standard characters. In addition, differences in PAUP* ‘standard DNA/RNA absolute’ differences with default settings (equating scheme in place; gaps treated as missing data) are given in square brackets. For those who wish to convert absolute to relative distances, alignment length was between 698–722 bp.

## Results and general discussion

Species recognition is often not easy in *Hebeloma*, and although species can normally be identified by morphology alone, species are delimited by a combination of morphology, multi-locus molecular data and ecology. In some sections (*H.* sects. *Denudata* and *Velutipes*) the efforts of Aanen and co-workers (i.e. Aanen and Kuyper 1999, 2004, Aanen and Kuyper 2004) also gave some evidence with regard to the limits of biological species. As described earlier (Eberhardt et al. 2015a, 2015b, 2016, Beker et al. 2016, Grilli et al. 2016), species definitions based on several lines of evidence may share ITS or other loci’ haplotypes, presumably as a result of incomplete linage sorting, hybridization or other population processes. The molecular distance between some species is so small that we assume that not all groups we recognize as species had sufficient time to reach monophyly in all loci. Thus, we do not necessarily expect species to form monophyla in ITS trees. In spite of this, and this is visualized by the networks, certain haplotypes or combination of haploypes (as in dikarya, here referred to as “variants”) is normally characteristic for a single species and occurs only rarely in sister species. Therefore, in spite of its lack of resolution in phylogentic trees, BLAST searches against an ITS database of well identified collections very often retrieve the correct species name in relation to other lines of evidence. We are not aware of a single locus that can differentiate between all species of *Hebeloma*. In particular in H.sect.Hebeloma, the search for a locus that is more powerful in recognizing species than the loci used by Beker et al. (2016), namely ITS, *RPB2*, *Tef1a*, and variable regions of the mitochondrial SSU, is still ongoing. We are at the beginning of our research into the *Hebeloma* funga of America and all of our conclusions rest heavily on our insights into *Hebeloma* of Europe and there on the available material. For some species, for example *H.velutipes*, we have hundreds of collections to choose from, while for other species, like *H.pubescens* we have only a few specimens. As our research goes on and more data becomes available, we will revisit and if necessary rectify the conclusions drawn here.

Sixteen species of *Hebeloma* were identified morphologically among the collections from the Rocky Mountains alpine zone. The molecular analysis carried out supported the morphological analysis. A key is given below. In all, 115 collections and 10 relevant types from North America were sequenced successfully for the ITS region (Tables 1, 2).

**Figure 2.**
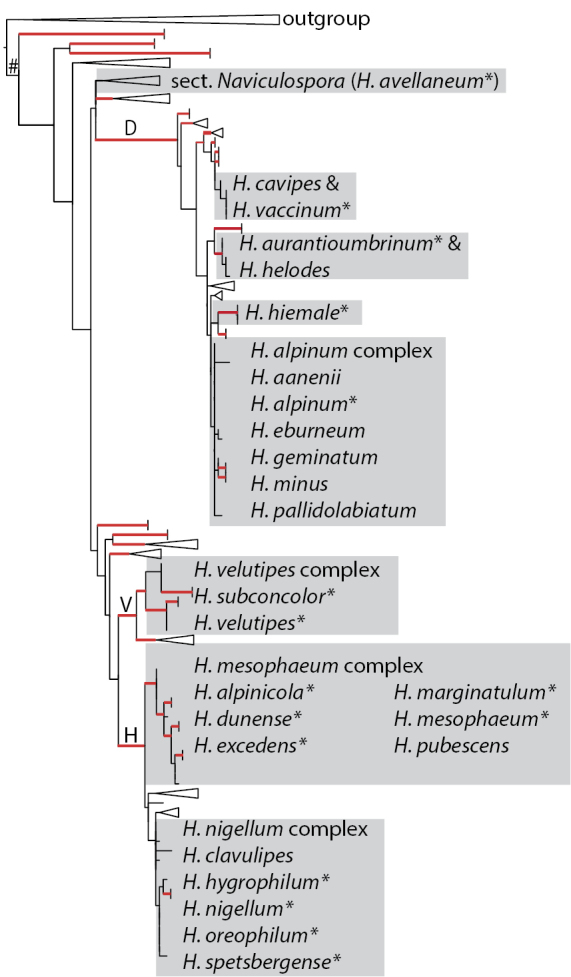
ITS overview tree of the genus *Hebeloma* in Europe from Beker et al. (2016) fig. 12A modified. Grey boxes indicate species clusters represented in separate tree or network figures. Red lines indicate branches with ML bootstrap support of ≥ 80%. # = genus *Hebeloma*; D = H.sect.Denudata; H = H.sect.Hebeloma; V = H.sect.Velutipes; * = species recorded from the Rocky Mountains. For further details see Beker et al. (2016) and the running text.

**Figure 3.**
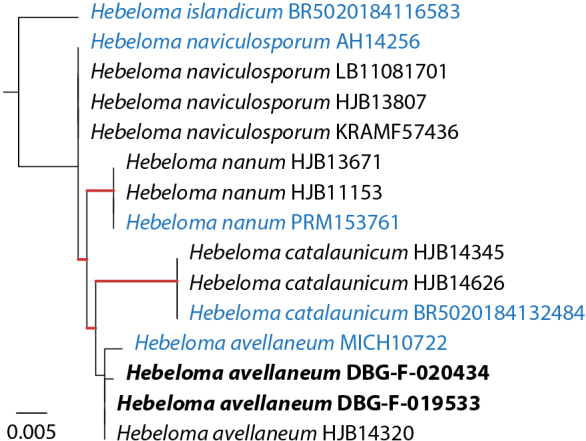
ML result of Hebelomasect.Naviculospora rooted in accordance with the results of Beker et al. (2016) with *H.islandicum* (internal outgroup). Branches supported by ≥ 80% bootstrap (1000 replicates) are indicated in red. Collections from the Rocky Mountains are indicated in bold, type sequences are indicated in blue.

**Figure 4.**
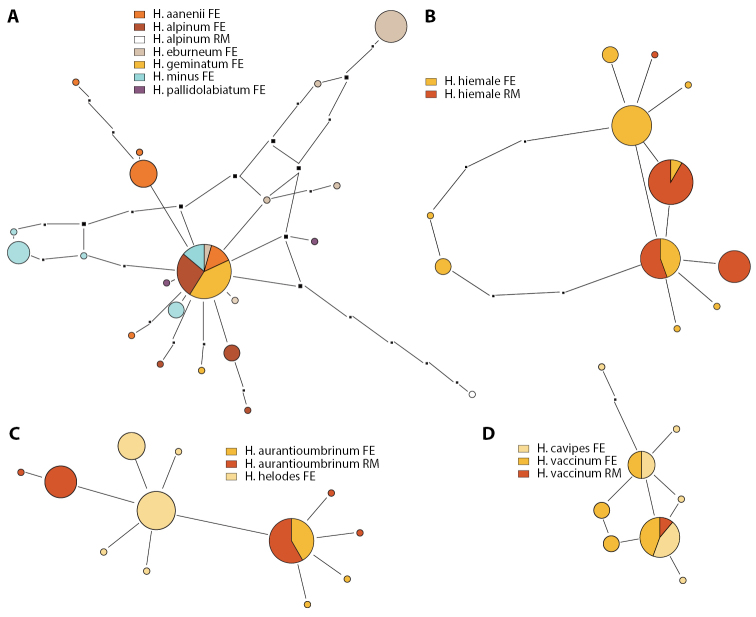
Pruned quasi-median networks of species and species clusters of Hebelomasect.Denudata. **A***H.alpinum* complex **B***H.hiemale***C***H.aurantioumbrinum* and *H.helodes***D***H.cavipes* and *H.vaccinum*. In networks, the size of the circles corresponds to the number of sequences they represent. Circles shared by two or more taxa are divided according to the number of representatives for each species. FE and RM refer to the origin of the collections, Europe or Rocky Mountains, respectively.

**Figure 5.**
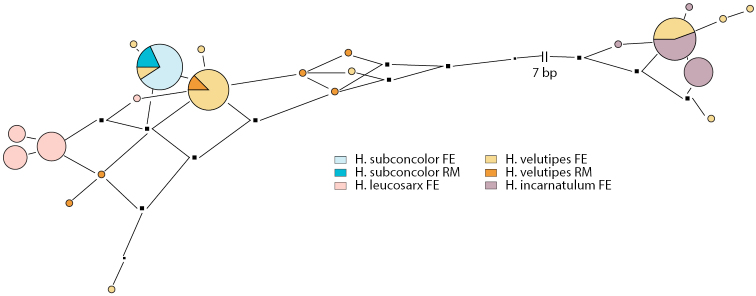
Pruned quasi-median networks of the *Hebelomavelutipes* complex. Circles shared by two or more taxa are divided according to the number of representatives for each species. FE and RM refer to the origin of the collections, Europe or Rocky Mountains, respectively.

**Figure 6.**
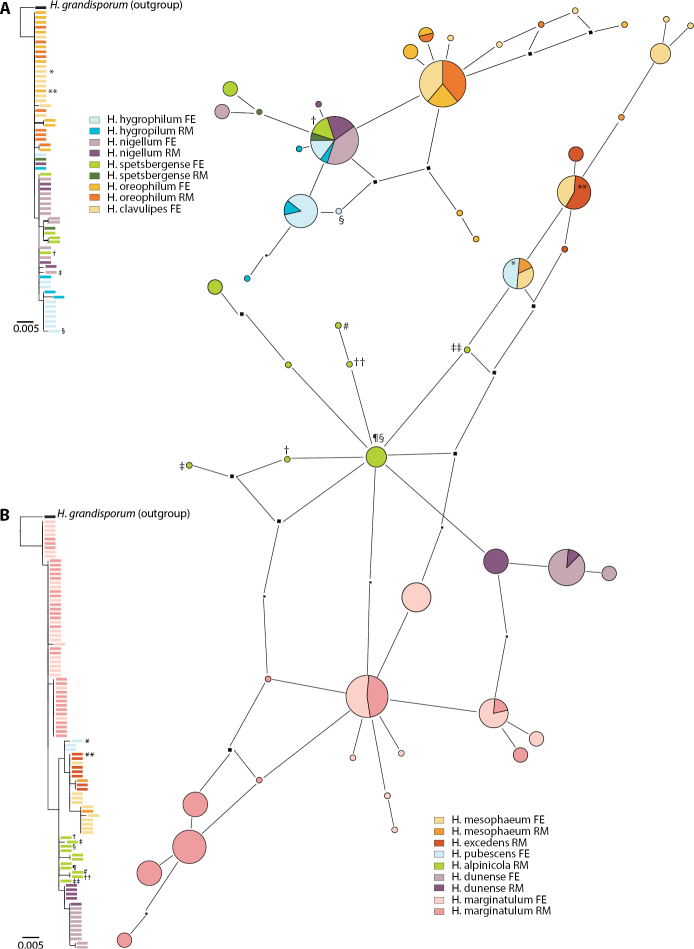
ML results and pruned quasi-median networks of species complexes of Hebelomasect.Hebeloma.**A***H.nigellum* complex **B***H.mesophaeum* complex. In ML trees, branches supported by ≥ 80% bootstrap (1000 replicates) are double width. In networks, the size of the circles corresponds to the number of sequences they represent. Circles shared by two or more taxa are divided according to the number of representatives for each species. FE and RM refer to the origin of the collections, Europe or Rocky Mountains, respectively. Placement of type sequences is indicated as follows: **A** * = *H.clavulipes.* ** = *H.oreophilum*, † = *H.spetsbergense*, ‡ = *H.nigellum* (not included in the network analysis), § = *H.hygrophilum*; **B** * = *H.pubescens*, ** = *H.excedens*, † = *H.subargillaceum*, ‡ = *H.nigromaculatum*, § = *H.littenii*, ¶ = *H.alpinicola*, # = *H.perigoense*, †† = *H.chapmaniae* and ‡‡ = *H.smithii*.

Figure 2 shows the taxonomic positions of the treated species (complexes) mapped on the ITS tree of Beker et al. (2016). Of the 16 species collected in the Rockies, three were not treated by Beker et al. (2016), namely *H.alpinicola*, *H.avellaneum* and *H.excedens*. These species were named based on type studies. Figure 3 shows that *H.avellaneum* is a member of H.sect.Naviculospora and forms a monophylum. The only other species encountered in the Rocky Mountains that is clearly distinct in the ITS region is *H.hiemale* (Beker et al. 2016; Eberhardt et al. 2016; Fig. 4B). For all other species, several taxa were included in a single network (Figs 4A, 4C, 4D, 5, 6). The networks show that there are usually only a small number of unambiguous base pair differences between members of the same species, irrespective of their origin, even though some parts of some networks (*H.aurantioumbrinum*, *H.marginatulum*) are exclusively of RM origin. While ITS trees were published for *H.* sects. *Denudata* and *Velutipes* (Eberhardt et al. 2015a, 2016; Grilli et al. 2016), this is not the case for H.sect.Hebeloma. Therefore ITS ML trees, rooted with *H.grandisporum* Beker, U. Eberh. & A. Ronikier, are shown in Figure 6. Details, including base pair (bp) differences between species, are discussed in the Taxonomy section.

Beker et al. (2016) showed that in a number of *Hebeloma* species clusters or complexes, morphology is better suited for species distinction and delimitation than molecular data. The majority of the species encountered in the Rocky Mountains belong to these species complexes. Thus, it is not surprising that the ITS analyses are only clear for two species, namely *H.avellaneum* and *H.hiemale*. For the other species, there is at least one other species with very similar ITS sequences. In some cases such as for *H.aurantioumbrinum* and *H.vaccinum*, the only sister taxa that cannot be distinguished by ITS sequence differ in habitat (Beker et al. 2016). Also, in the larger complexes, not all of the considered species are associated with the same hosts or habitats as the target species. *Hebelomaclavulipes* Romagn., *H.eburneum* Malenҁon, *H.incarnatulum* A.H. Sm., and *H.leucosarx* P.D. Orton are not expected to occur in the habitats sampled in the Rocky Mountains; *H.aanenii* Beker, Vesterh. & U. Eberh. and *H.geminatum* Beker, Vesterh. & U. Eberh. hardly ever grow in such habitats (Beker et al. 2018).

In the Taxonomy part, minute levels of sequence variation are discussed. We do that against the background of multilocus analyses presented by Beker et al. (2016) and other works, indicating in which cases the ITS is wanting for species differentiation. Thus, even though ITS differences between species may be slight or not constant, and even considering that morphological distinction in some cases relies on minute differences, the combination of morphology, ecology, and ITS data provides a reliable set of information for species assignment.

Based on previous studies, delimitation of most species is now well understood (Eberhardt et al. 2015a, 2016; Beker et al. 2016; Grilli et al. 2016), and consequently we did not consider it necessary to include all species discussed as morphologically similar in the same molecular analysis. Our aim has been to show what information, even in the case when it is sparse, is contained in ITS data.

We have made an effort to combine sequence analyses based on different subsets of data and displaying different levels of complexity in the visualization. We have considered several different methods for analyzing ITS sequence data: ML trees, pruned quasi-median networks, and base pair difference counts between aligned sequences. Sometimes, the relationship between sequences and species may appear differently between trees, networks and difference counts. In the ML analyses, gaps are treated as missing data and ambiguous reads are equated. The networks are based on clean base pair exchanges and gaps; polymorphic positions with two states, i.e. positions with ambiguous codes are treated as missing data. Owing to the complexity of networks displaying this kind of information in full, such networks are, as far as we are aware, used for data verification rather than for data analysis (Bandelt and Dürr 2007; Brandstätter et al. 2007). For the direct sequence difference counts, all kinds of differences were counted equally, thus giving the maximum number of differences plus giving absolute DNA differences in square brackets, which do not count gaps and polymorphic positions as different. Whereas ML trees pruned quasi-median networks and absolute DNA differences are prone to omitting observed intragenomic and thus intraspecific variation, total distance counts are overestimates. In spite of that, we have decided to present these values here, because they could influence species identificaton.

### Key to *Hebeloma* species of the Rocky Mountain Alpine Zone

**Table:** 

1	Cortina absent; pileus mostly uniform in color, lamellae often with droplets; stipe base usually not dark; cheilocystidia mostly clavate or capitate (swollen near the apex, sometimes also in the lower half); spores mostly amygdaliform	**2**
2	Pileus small, 10–20(–25) mm, stipe 2–4 mm wide; and with 20–40 full length lamellae	**3**
3	Spores on ave. at least 12 µm long, distinctly finely verrucose, dextrinoid; pileus brown, reddish brown; stipe cream; with *Salix*	**1. *H.vaccinum***
3*	Spores on ave. <12 µm long, not or weakly ornamented, slightly dextrinoid; pileus a different color	**4**
4	Pileus uniformly pinkish buff, orange brown; margin crenate with white rim; stipe whitish; cheilocystidia significantly constricted below the apex, ave. median width at most 5 µm; with *S.planifolia* or *S.arctica*	**2. *H.aurantioumbrinum***
4*	Pileus brown, grayish brown, pruinose; stipe buff; cheilocystidia tapering more gently towards base, ave. median width at least 5 µm; with *Salix*	**3. *H.subconcolor***
2*	Pileus larger, 20–60 mm; stipe wider 5–15 mm; and with 40–100 full length lamellae	**5**
5	Spores distinctly verrucose, not or weakly dextrinoid, on ave. 10–12.5 × 5–7 µm; cheilocystidia swollen at apex and also in the lower half; pileus cream, pinkish buff, isabella; stipe clavate, floccose; mostly with *S.reticulata* in the Rockies	**4. *H.hiemale***
5*	Spores only slightly rough, weakly to strongly dextrinoid	**6**
6	Pileus rich brown, orange brown, cinnamon brown, margin rolled under; lamellae pale, stipe whitish; odor fruity; spores on ave. 8.5–10 × 5–5.5 µm, narrow, distinctly dextrinoid; in lower alpine with conifers (poss. *Salix*)	**5. *H.avellaneum***
6*	Pileus paler; spores somewhat larger; with *Dryas* or dwarf *Salix*	**7**
7	Pileus pale buff, pinkish buff; stipe stout, white, half floccose, often long, often with bulbous base; often with *Dryas* in the Rockies alpine; spores moderately to strongly dextrinoid	**6. *H.velutipes***
7*	Pileus cream to pale brown, robust; stipe mostly equal, shorter; with *Dryas* or *Salix*; spores at most weakly dextrinoid	**7. *H.alpinum***
1*	Cortina present; pileus often two-colored, with darker center and paler margin; lamellae not or minimally weeping; stipe often black or dark at base; cheilocystidia lageniform to ventricose (swollen in lower half); spores elliptical or amygdaliform	**8**
8	Spores elliptical; rather smooth, not dextrinoid; slightly larger types with wider stipes (typically 4–8 mm); with *Salix* spp	**9**
9	Pileus with darker coloration, brown, reddish brown	**10**
10	Pileus dark brown, hoary; lamellae deeply emarginated; margin turned in and coated with veil remnants; spores on ave. at least 10 × 6 µm	**8. *H.marginatulum***
10*	Pileus robust, reddish brown with grayish cast; stipe stout, base often encased in sand, cespitose; spores on ave. <10 µm long and <6 µm wide	**9. *H.alpinicola***
9*	Pileus with paler coloration, pinkish buff, light brown, yellowish brown, can be dark in center	**11**
11	Spores on average at least 10 × 6 µm, slightly ornamented; pileus pinkish buff, brown, hoary, more unicolor; lamellae subdecurrent or sinuate; yellow contents in some cystidia; with dwarf willows or *S.planifolia*	**10. *H.dunense***
11*	Spores on ave. <10 µm long, almost smooth; with *Salixglauca* in alpine Rockies	**12**
12	Pileus ocher, darker in center, two-toned	**11. *H.mesophaeum***
12*	Pileus pale brown, pinkish brown, more uniform; margin can exceed lamellae	**12. *H.excedens***
8*	Spores amygdaliform, finely verrucose, dextrinoid; smaller types with thinner stipes, 1–4(–8) mm in diam.; mostly with *S.planifolia*	**13**
13	Pileus 20–40 mm, brown, lamellae >40, stipe 3–8 mm wide; in moss or not; spores on ave. 11–14 × 6.8–7.2 µm	**13. *H.oreophilum***
13*	Pileus 8–25 mm, pale brown with blackish brown center; lamellae <40; stipe thin, 1–4 mm wide; typically in moss	**14**
14*	Spores on ave. 11.4 × 6.8 µm wide; epicutis >100 µm thick	**14. *H.hygrophilum***
14*	Spores on ave. 11.9 × 7.2 µm; epicutis less than 100 µm thick	**15. *H.nigellum***
14**	Spores on ave. at least 7.5 µm wide; on av 12.5 × 7.6 µm	**16. *H.spetsbergense***

## Taxonomy

### Descriptions of Rocky Mountain Collections

Descriptions of Rocky Mountain *Hebeloma* species 1–16 are presented in the order shown in the key for convenient access.

### *Hebeloma* sections *Denudata* (Fr.) Sacc., *Velutipes* Vesterh., and *Naviculospora* Beker & U. Eberh. – species without a cortina.

#### 1. Hebeloma vaccinum

Taxon classificationFungiAgaricalesHymenogastraceae

Romagn., Bull. Trimest. Soc. Mycol. Fr. 81: 333 (1965)

**Figure 7.**
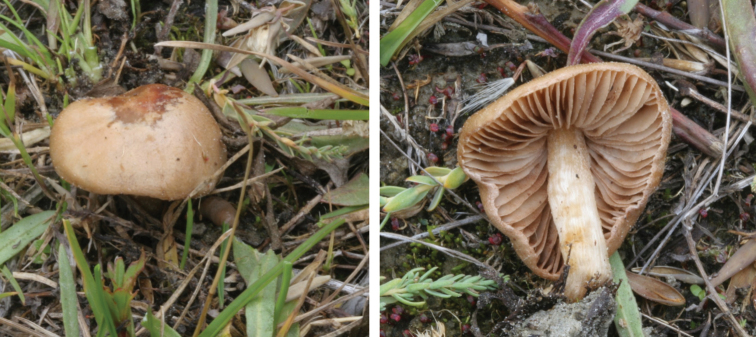
*Hebelomavaccinum* HJB11135 from Swiss alpine zone.

Figures 4D , 7 , 23 (1)

##### Etymology.

From *vaccinus*, meaning dun color (i.e. dull grayish brown).

##### Description.

Cortina not observed. Pileus 10–11 mm in diameter, convex, buff to brownish with a hoary coating, rather unicolor, smooth, shiny, tacky; margin turned down, a bit crenulate, faintly striate; edges white. Lamellae adnexed, L = 38 plus lamellulae, buff to milk coffee. Stipe 10 × 3 mm, equal, cream, finely floccose at apex and fibrillose for length, delicate. Context cream. Odor not apparent, but previously noted as raphanoid. Exsiccate: very tiny, brown, not shiny, lamellae not blackening.

Basidiospores yellowish brown, amygdaliform, limoniform, with a snout and small apiculus, distinctly verrucose (O3), with loosening perispore observed in a few spores (P1, P2), dextrinoid (D3), 10–14 × 6–8 µm, on average 12.2 × 7.1 µm, Q = 1.71; some larger spores present –18 × –9 µm. Basidia 27–35 × 7–9 µm, four-spored, possibly a few two-spored because of larger spores present. Cheilocystidia clavate-lageniform, some slightly more swollen at apex, 35–70 × 6–8 µm at apex, 3–5 µm in middle, and 6–10 µm at base, occasionally septate, no thickening observed. Pleurocystidia absent. Epicutis thickness 40–125 µm, with some encrusted hyphae.

##### Rocky Mountain Ecology

results are based on a single collection of two small basidiomes found in the Colorado alpine with *Salixarctica*.

##### Rocky Mountain specimen examined.

U.S.A. COLORADO: San Juan County, San Juan Mountains, Mineral Basin, with *Salixarctica*, 3320 m, 31 July 2002, CLC1881 (MONT), C. Cripps.

##### Discussion.

Beker and co-workers (Beker et al. 2016; Eberhardt et al. 2016; including ML ITS analyses) showed that *H.vaccinum* can be recognized by its ITS region from all species apart from *H.cavipes* Huijsman, which differs in morphology and ecology. The RM *H.vaccinum* collection fits in with the diversity found within the species (Fig. 4D) it differs in 0–5 [0] bp from other included members of the species. The intraspecific variation of the included FE members of *H.vaccinum* is 0–8 [0] bp.

This species is usually described as larger (13–40 mm) than the Rocky Mountain specimens described here. Microscopically, the species has spores that are strongly dextrinoid (D3) with a frequently loosening perispore. The spores and cheilocystidia characteristics (swollen at the apex and at the base but constricted in the middle part) put it in H.sect.Denudata, subsect. *Clepsydroida. Hebelomavaccinum* is known to occur in low elevation dunes and woodlands with *Salix*; it is widespread in Northern Europe. Other arctic-alpine collections are from the European Alps, the Carpathians in Slovakia, and Greenland, always with *Salix* species (Beker et al. 2016; Eberhardt et al. 2015b). It could be recognized in the Rocky Mountains by its association with dwarf *Salix*, small size, lack of a veil, and distinct spores and cystidia; compare with *H.aurantioumbrinum*.

#### 2. Hebeloma aurantioumbrinum

Taxon classificationFungiAgaricalesHymenogastraceae

Beker, Vesterh. & U. Eberh., Persoonia 35: 116 (2015)

**Figure 8.**
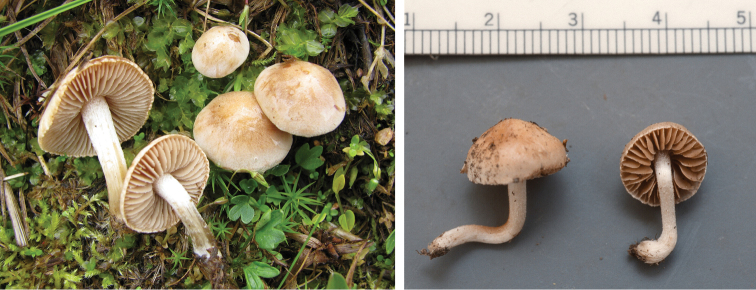
*Hebelomaaurantioumbrinum*, CLC3093 and CLC1822.

Figures 4C , 8 , 23 (2)

##### Etymology.

From *aurantius*, orange and *umbrinus*, umber.

##### Description.

Cortina absent. Pileus small, 10–20 mm in diameter, convex, slightly conic-convex, appearing smooth, greasy, not hygrophanous, cream, then buff, pinkish buff, orange brown, can be lighter towards margin but not clearly two-toned, somewhat hoary; margin weakly involute, possibly crenate with a white rim. Lamellae deeply indented, deeply sinuate-arcuate, rather distant, L = 25–40 plus lamellulae, cream, then buff, pinkish buff, milk coffee; edges fimbriate, white but graying, drops visible. Stipe 15–28 × 2–3 mm, equal, bit curved, dingy whitish cream but darkening at base to watery brown (in CLC3093), floccose/pruinose for top third and smooth-fibrous below. Context dingy whitish. Odor faint or raphanoid. Exsiccate: pileus buff, lamellae brown; stipe very thin, whitish.

Basidiospores yellowish brown, slightly amygdaliform, with almost obtuse ends, with tiny apiculus, with slight ornamentation (O2), no loosening perispore (P0, P1), slightly dextrinoid (D1, D2), 10–13(–14) × 6–7.5 µm, on average 11.5 × 6.7 µm, Q = 1.72. Basidia 30–35 × 8–10 µm, clavate, two- and four-spored. Cheilocystidia long with swollen apex, clavate-stiptate, occasionally clavate-lageniform, 40–70 × 6–9 µm at apex, 3–5.5 µm in middle, and 3–6.5 µm in base. Pleurocystidia absent. Epicutis thickness 70–100 µm, with some encrusted hyphae.

##### Rocky Mountain ecology.

In the alpine with willows *Salixglauca*, *Salixplanifolia*, and *S.arctica*, reported from Colorado, Montana and Wyoming.

##### Rocky Mountain specimens examined.

U.S.A. COLORADO: San Juan/Hinsdale County, San Juan Mountains, Stony Pass, with *Salixarctica*, 28 July 2002, CLC1822 (MONT), C. Cripps. WYOMING: Park County, Beartooth Plateau. Frozen Lakes with *S.planifolia*, 14 Aug 2014, CLC3093 (MONT), C. Cripps; WY/MT stateline with *S.planifolia*, 14 July 2001, CLC1565 (MONT), C. Cripps. Wyoming Creek 6 Aug 2008 with *S.arctica* and *S.glauca*, HJB12445, C. Cripps & H.J. Beker; HJB12446, C. Cripps; HJB12447, C. Cripps; HJB12448, H.J. Beker; HJB12450, HJB12452, HJB12453, H.J. Beker; HJB12451 with *S.planifolia*, H. Knudsen; HJB12454, E. Horak. Upper Wyoming Creek, with *Salixarctica*, 8 Aug 2008, HJB12456, J. Antibus. Hell-Roaring Plateau, with *Salix* sp., 14 Aug 2007, ZT12730 (ETH), ZT12731 (ETH), E. Horak.

##### Discussion.

Beker and co-workers (Beker et al. 2016; Eberhardt et al. 2015a) showed that *H.aurantioumbrinum* cannot be distinguished from the non-arctic-alpine *H.helodes* J. Favre based on ITS sequencing, but it can be separated from all other members of H.sect.Denudata. An ITS tree is given in Eberhardt et al. (2015a). The RM dataset includes more collections of *H.aurantioumbrinum* (15) than the FE dataset (7). Therefore, it is not surprising that the molecular diversity of the RM sequences is higher than that of the FE dataset (Fig. 4C). There are 0–6 [0] bp differences among the FE sequences of *H.aurantioumbrinum*, 0–9 [0–3] bp differences among the sequences of RM *H.aurantioumbrinum* and 2–11 [0–3] bp differences between *H.aurantioumbrinum* and *H.helodes*. Morphologically, *H.aurantioumbrinum* and *H.helodes* are quite different and can be easily separated, for example *H.helodes* always has a distinct thickening of the cheilocystidium wall at the apex, a feature that is absent in *H.aurantioumbrinum*. Further, they occur in very different habitats; *H.helodes* has never, to our knowledge, been confirmed in arctic-alpine habitats.

*Hebelomaaurantioumbrinum* may have been confused with *H.pusillum* J.E. Lange, although *H.pusillum* has much more slender basidiomes that are distinctly two-toned. *Hebelomaaurantioumbrinum* is squatter and rarely two-toned. Additionally, we are not aware of any confirmed records of *H.pusillum* in arctic-alpine habitats. Both these species, without any veil (beyond the primordial stage) and with clavate-stiptate cheilocystidia, belong to the Crustuliniformia subsection of section Denudata. This subsection contains many small species that are arctic-alpine specialists that occur with *Salix*, and these species have only recently been split out and described (Eberhardt et al. 2015a). Collections of *H.aurantioumbrinum* have been confirmed from a number of arctic and alpine habitats, including Greenland, Iceland, Scandinavia, and Svalbard (Beker et al. 2016). In the Rockies, this species can be recognized by its alpine habitat, association with willows (primarily *S.planifolia*), small size, lack of veil, and pinkish buff to orange brown uniformly colored pileus often with a white, crenate margin.

#### 3. Hebeloma subconcolor

Taxon classificationFungiAgaricalesHymenogastraceae

Bruchet, Bull. Mens. Soc. Linn. Lyon 39 (6, suppl.): 127 (1970)

**Figure 9.**
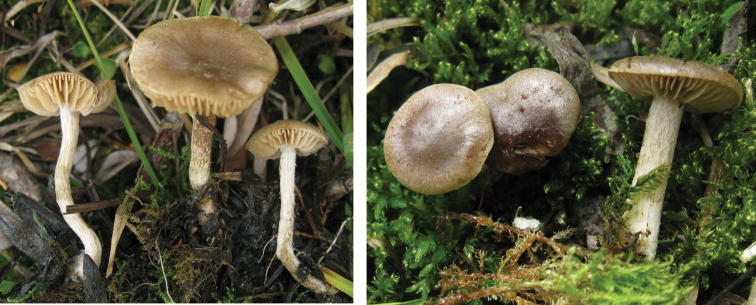
*Hebelomasubconcolor*, DBG-F-022785 and DBG-F-022786.

Figures 5 , 9 , 23 (3)

##### Etymology.

*concolor* for the similar coloration of pileus and stipe, which is not a consistent feature.

##### Description.

Cortina absent. Pileus 15–20 mm, convex, with or without a low broad umbo, becoming plane, smooth, moist, light to medium brown, pruinose with a grayish tint or sheen, lighter towards margin but not distinctly two-toned; margin turned down or not, entire. Lamellae adnexed, subdistant, well-separated, medium broad to broad, L = 25–32 plus lamellulae, dull brown, light brown; edges lighter. No beaded drops reported. Stipe 15–30 × 3–4 mm, equal, apex somewhat lighter tan and pruinose, below totally covered with longitudinal white fibers over a brownish ground base. Context buff. Odor astringent. Exsiccate: pileus medium brown, not two-toned, with grayish tint, dull; lamellae broad, warm cinnamon; stipe long, dull brown, narrow.

Basidiospores yellowish brown, amygdaliform, with a small apiculus, weakly ornamented (O2), loosening perispore observed in a few spores (P0, P1), distinctly dextrinoid (D2, D3), 10.5–12.5 × 6.5–7.5 µm, on average 11.6 × 7.1 µm, Q = 1.65. Basidia 25–34 × 8–10 µm, four-spored. Cheilocystidia gently clavate, some slightly swollen at apex and base, 40–60 × 6–11 µm at apex, 4.5–7 µm in middle, and 4–7 – (8) µm at base. Pleurocystidia absent. Epicutis thickness 60–75 µm, with some encrusted hyphae.

##### Rocky Mountain ecology.

Two collections reported under willow at alpine elevations of 4000 m in Colorado; noted as cespitose to gregarious.

##### Rocky Mountain specimens examined.

U.S.A. COLORADO: Clear Creek County, Summit Lake Park, under *Salix*, some in moss, at 4000 m, 22 Aug 2012, DBG-F-022785; DBG-F-022786, L. Gillman.

##### Discussion.

The sequences of the two collections for *H.subconcolor* from the Rocky Mountains are identical. The RM sequence differs by 1–4 [0] bp from the *H.subconcolor* collections described in Beker et al. (2016) and Grilli et al. (2016), where the ITS ML results were also shown. The closest *H.velutipes* sequence included in the dataset used in Fig. 5 differs in 3 [0] bp. *Hebelomavelutipes* is the only species of *Hebeloma* that cannot be distinguished from *H.subconcolor* by ITS sequence (Beker et al. 2016; Grilli et al. 2016; Fig. 5). However, morphologically these two species are very different and can be easily separated.

This small species has a grayish cast not found in other taxa in sections *Denudata* and *Velutipes* that we report from the Rocky Mountains; also, the lamellae are well separated and few in number. It should be compared to the other non-veiled, small species such as *H.aurantioumbrinum* and *H.vaccinum*. *Hebelomavelutipes* has a different coloration and is larger with many more full length lamellae. *Hebelomasubconcolor* is known from arctic and alpine locations in the European Alps, Greenland, Iceland and Scandinavia (Beker et al. 2016, 2018).

#### 4. Hebeloma hiemale

Taxon classificationFungiAgaricalesHymenogastraceae

Bres., Fung. Trident. 2: 52 (1898)

**Figure 10.**
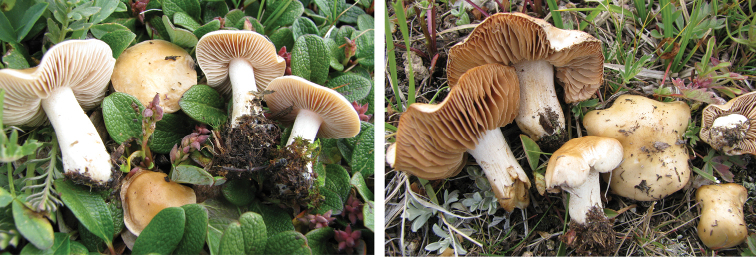
*Hebelomahiemale*, CLC3094 and CLC3574.

Figures 4B , 10 , 23 (4)

##### Etymology.

From *hiemalis*, winter or wintry, presumably to denote the production of basidiomes in colder seasons or habitats

##### Description.

Cortina absent. Pileus 15–35 mm in diameter, slightly conic-convex or domed-convex, smooth, greasy, pinkish buff, yellowish buff, to pale cream at the margin, with uniform coloration, somewhat hoary, with or without a white rim a few mm wide at margin; margin turned down or rolled in, then wavy. Lamellae narrowly attached, emarginate, somewhat crowded, L = 48–60 plus lamellulae, white to pale milk coffee, pale brown, wood brown; edges white floccose, with drops of liquid. Stipe 20–45 × 5–12 mm, equal, slightly clavate towards the base, whitish cream, totally pruinose (big floccules) for most of length and smoother below. Context white to watery cream, firm. Odor raphanoid, faint. Exsiccate: pileus yellowish brown, not distinctly two-toned; lamellae brown with white edges; stipe white and slimmer than for *H.alpinum*.

Basidiospores yellowish brown, some coloring slightly brown in Melzer’s, fat-bellied amygdaliform, limoniform, with short snout, apiculate, distinctly ornamented (O2), a few with slightly loosening perispore (P0,P1), rarely guttulate, with thickish wall, slightly dextrinoid (D1, rarely D2), 10–12 × 6–7 µm, on average, 11.1 × 6.8 µm, Q = 1.64. Basidia 25–35 × 7–9, most four-spored, maybe a few two-spored, occasionally with long sterigmata (–5 µm). Cheilocystidia long, gently clavate, clavate-lageniform, some with septa, 35–75 µm long, at apex 6–9 µm, in middle 4–6 µm, at base 4.5–9 µm, thickening sometimes observed in the middle. Pleurocystidia absent. Epicutis thickness 60–200 µm, with some encrusted hyphae.

##### Rocky Mountain ecology.

In the alpine zone with dwarf willows, *Dryas* and *Betula*, confirmed from Colorado, Montana, and Wyoming.

##### Rocky Mountain specimens examined.

U.S.A. COLORADO: Summit County, Loveland Pass, 3750 m, with *Salix* in scrubland, 7 Aug 1999, ZT8072 (ETH), E. Horak; 15 Aug 1997, 3655 m, with *Salix*, DBG-F-019162, B. Rognerud; 21 Aug 2003, with *Salix* sp., DBG-F-021418, H. Miller; 20 Aug 1999, 3597 m, with *Betula*, DBG-F-020440, O.K. Miller; 22 Aug 1999, 3655 m, with *Salix* sp., DBG-F-020437, O.K. Miller; 16 Aug 1997, 3749 m, with *Salix* sp., DBG-F-019241, S. Trudell; 19 Aug 1999, 3620 m, DBG-F-021194, V.S. Evenson; 20 Aug 1999, 3620 m, with *Salix* sp., DBG-F-20431, V.S. Evenson; 20 Aug 1999, 3571 m, with *Betulanana*, DBG-F-020433, V.S. Evenson; 24 Aug 1999, 3620 m, with *Salix* sp., DBG-F-019597, N. Smith Weber; Clear Creek County, Mount Goliath, 3658 m, with *Salix*, 1 Sept 1999, DBG-F-020551, V.S. Evenson; 1 Sept 1999, 3810 m, DBG-F-020550, V.S. Evenson; Boulder County, West of Caribou townsite, 10 July 1988, DBG-F-016104, V.S. Evenson. Sawatch Range, Independence Pass, 13 Aug 2001, 3759 m, with *Dryasoctopetala* and *S.reticulata*, ZT9828, E. Horak. San Juan County, San Juan Mountains, Mineral Basin, 3835 m, with *Salixarctica*, 7 Aug 2001, CLC1668 (MONT), C. Cripps. MONTANA: Carbon County, Beartooth Plateau (at the stateline with WY), 3100 m near *S.reticulata*, 19 July 2001, CLC1574 (MONT), C. Cripps; site 2 at the stateline MT/WY, with *S.reticulata* 14 Aug 2014, CLC3094 (MONT), C. Cripps; Quad Creek, 3004 m, with *S.reticulata* and *Persicariavivipara*, 8 Aug 2008, HJB12457, M. Nauta; site 1 in *Dryas*, 11 Aug 2017, CLC3533 (MONT), C. Cripps; with *S.planifolia* and *S.glauca*, 17 Aug, 2017, CLC3574 (MONT), C. Cripps; with *Salixplanifolia*, 17 Aug 2017, CLC3575 (MONT), C. Cripps. WYOMING: Park County, Highline Trail, 3200 m, with *Dryasoctopetala* and *S.reticulata*, 8 Aug 2008, ZT6417 (ETH), E. Horak.

##### Discussion.

An ITS tree including *H.hiemale* is given by Eberhardt et al. (2016); the respective network is shown in Figure 4B. The RM dataset includes ITS sequences from 22 collections. These were matched by the same number of sequences from the FE dataset. *Hebelomahiemale* ITS sequences were shown to form a well-supported monophylum in ML results presented in earlier studies (Beker et al. 2016; Eberhardt et al. 2016). Beker et al. (2010) showed that it is a species with a relatively high number of different ITS variants. The disparity between variants is mostly caused by gaps and SNPs (single-nucleotide polymorphisms). The number of differences between any pair of sequences of the presented *H.hiemale* data set is 0–9 [0–2] bp, within the RM sequences 0–8 [0] bp.

This species is widespread across Europe occurring from the subalpine to the alpine, in lowland dunes, shrublands, gardens, and parks; it occurs with a wide array of deciduous and coniferous trees and this includes a number of willow species, including dwarf *Salix*. Confirmed arctic-alpine reports include those from Canada, Greenland, Iceland, Scandinavia, and Svalbard with *Salixherbacea* and *S.polaris* as well as *Dryas* and *Persicaria* (Beker et al. 2016). Here it is confirmed with *S.reticulata*. *Hebelomahiemale* has rarely been reported from North America in either subalpine or alpine habitats (Beker et al. 2010), but many collections previously labeled *H.alpinum* are now confirmed as *H.hiemale*.

This species looks like a small version of *Hebelomacrustuliniforme* but usually has more color in the pileus, particularly at the center. It has cheilocystidia that are generally swollen in the lower half, giving an hourglass appearance. The spores are verrucose, more warty than those of *H.alpinum*, but less so than the spores of *H.vaccinum*. There was some ambiguity around the delineation of *H.hiemale*, which was ultimately resolved with selection of an epitype (Beker et al. 2010; Eberhardt et al. 2015a).

#### 5. Hebeloma avellaneum

Taxon classificationFungiAgaricalesHymenogastraceae

Kauffman, Papers of the Michigan Academy of Sciences 17: 171 (1933)

**Figure 11.**
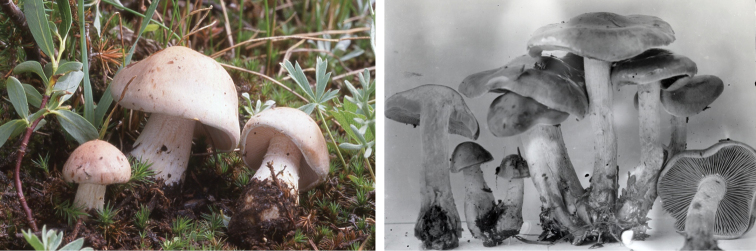
*Hebelomaavellaneum*, DBG-F-019533 and UMICH 10722 (holotype).

Figures 3 , 11 , 23 (5)

##### Etymology.

For the color of hazelnuts, such as *Corylusavellana*.

##### Description.

Cortina absent. Pileus 20–40 mm across, hemispherical, convex, can be domed, glabrous-viscid, rich Sayal brown, ochraceous to orange brown, cinnamon brown, with frosty canescence; margin turned down, or rolled in, remaining light colored, downy. Lamellae adnate to subdecurrent, narrow, L = 90 plus lamellulae, pale avellaneous, pale cinnamon, not dark at maturity; edges floccose, beaded. Stipe 25–35 mm × 8–10 mm, equal to clavate, sturdy, white to cream, pruinose at apex, scurfy scales below. Context thick over pileus area, whitish, watery, not changing, or browning a bit in stipe but not from base up. Odor fruity or herbal tones. Exsiccate: medium-sized, cespitose in one group, hemispherical with margin inrolled, evenly colored, ochraceous, smooth to aereolate; stipe white, sturdy.

Basidiospores yellowish brown, amygdaliform, with a small apiculus, weakly ornamented (O1, O2), loosening perispore observed in a few spores (P0, P1), distinctly dextrinoid (D3), 8–11 × 5–6 µm, on average 9.5 × 5.4 µm, Q = 1.76. Basidia 25–34 × 6.5–8.5 µm, two- and four-spored. Cheilocystidia variable, many cylindrical, but also gently clavate, capitate and capitate-stipitate as well as clavate-lageniform, 30–80 × 4–13(–15) µm at apex, 3.5–6.5 µm in middle, and 4–8(–9) µm at base. Pleurocystidia absent. Epicutis thickness 80–130 µm, no encrusted hyphae recorded.

##### Rocky Mountain ecology.

Cespitose, or clustered, in low alpine krummholz with conifers and willows. Both collections we have studied are from Colorado.

##### Rocky Mountain specimens examined.

U.S.A. COLORADO: Summit County, Loveland Pass Lake, 4000 m, under willows, 20 Aug 1999, DBG-F-020434, no conifers mentioned but present in the general area, O.K. Miller Jr; Boulder County, above Mountain Research Station, 3200 m, with small willows (*Salixplanifolia*) and one spruce within 2 m, 1 Aug 1998, DBG-F-019533, V.S. Evenson.

##### Other American specimens examined.

U.S.A. WASHINGTON: Grays Harbor County, Lake Quinault, Olympic National Park, at 75 m, on mossy edge of forest clearing, 8 Nov 1925, MICH 10722, C.H. Kauffman (**holotype**). CANADA. NEWFOUNDLAND AND LABRADOR: Pinware River at 15 m, under conifers, 7 Sep 2005, HJB14320, leg. J. May.

##### Discussion.

Based on ITS data, *H.avellaneum* is monophyletic, but unsupported by bootstrap values (Fig. 3). In terms of phylogeny, its closest relative is *H.catalaunicum* Beker, U. Eberh., Grilli & Vila, a Mediterranean species. It is also close to *H.naviculosporum* Heykoop, G. Moreno & Esteve-Rav. and *H.nanum* Velen. All three species appear to associate with *Pinaceae* (Beker et al. 2016). The identification of *H.avellaneum* is supported by type studies. The fourth collection used in Fig. 3 is from Canada (Newfoundland) and has been presented by Voitk et al. (2016) as “Hebelomasp.sect.Naviculospora”.

Based on our studies of this taxon and of the habitats where it has been collected, we strongly suspect that this species is typically associated with conifers in temperate to subalpine or subarctic habitats. The holotype was collected in a temperate rainforest within the Olympic Peninsula in western Washington state. The often pruinose pileus with distinctive orange tones is indicative of H.sect.Naviculospora. These specimens were found in the low alpine where conifers are possible, and indeed *Picea* was noted for one collection, but only willows for the other. In the low alpine of the Rocky Mountains, the species might be confused with *H.alpinum*, *H.velutipes*, or *H.hiemale* because of its robust habit and lack of veil, however there are more orange color tones of the pileus; the spores are smaller and more dextrinoid than one would expect for *H.alpinum* and *H.hiemale*.

#### 6. Hebeloma velutipes

Taxon classificationFungiAgaricalesHymenogastraceae

Bruchet, Bull. Mens. Soc. Linn. Lyon 39 (6, suppl.): 127 (1970)

**Figure 12.**
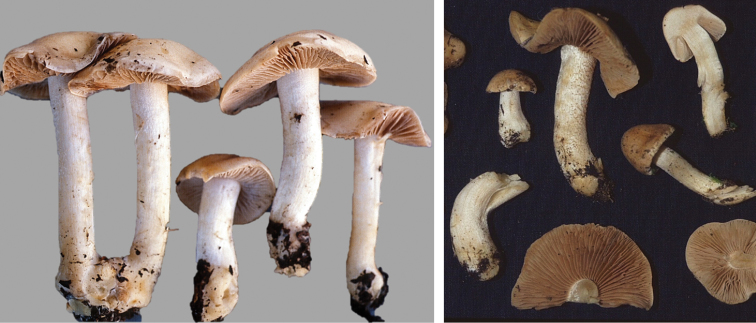
*Hebelomavelutipes*, CLC1651 and ZT8072.

Figures 5 , 12 , 23 (6)

##### Etymology.

*velutinus*, for the velvety appearance of the stipe surface.

##### Description.

Cortina absent. Pileus 20–60 mm in diameter, convex, convex-domed, tacky to kidskin, smooth, not spotting, not hygrophanous, nearly unicolor, very pale buff, pale salmon buff, with hoary coating (pruinose); margin incurved but not involute, entire. Lamellae narrowly attached, sinuate or marginate, narrow to broad, slightly crowded, L = 50–75 plus lamellulae, white at first, then milk coffee color; edges white-floccose; beaded drops observed on some. Stipe (25–)30–60 × 7–15 mm, robust, equal and either narrowing or swollen at base up to 20 mm wide, slightly curved or not, pruinose or floccose in top half, longitudinally fibrous in lower half or more smooth. Context whitish, thick in pileus, firm in stipe, stuffed/hollow. Odor raphanoid. Exsiccate: largest of all species recorded; uniform pale buff pileus, lamellae, and stipe.

Basidiospore print deep Sayal brown. Basidiospores yellowish brown, amygdaliform, with a slight snout, apiculate, not guttulate, a bit rough (O1, O2), moderately dextrinoid (D2, D3), no obvious loosening perispore (P0), 10–12 × 6–7 µm, on average 10.4 × 6.6 µm, a few large spores (–18 × –7) present, Q = 1.57. Basidia 26–32 × 7.5–9 µm, clavate, four-spored. Cheilocystidia gently clavate, thin-walled, occasionally bifurcate at apex, 55–80 µm × 7–12 µm at apex, 5–8 µm in middle, 4–7 µm at base. Pleurocystida absent. Epicutis thickness 80–200 µm, with some encrusted hyphae.

##### Rocky Mountain alpine ecology.

In alpine situations, mostly reported with *Dryasoctopetala* and also with *Salix* in Montana and Colorado.

##### Rocky Mountain specimens examined.

U.S.A. COLORADO: Gunnison County, Sawatch Range: Cumberland Pass, 3660 m, near *Salixglauca* but *Dryas* in vicinity, 4 Aug 2001, CLC1651 (MONT), C. Cripps; Cottonwood Pass, 3700, in pure *Dryasoctopetala*, 4 Aug 2001, CLC1646 (MONT), 12 Aug 2001, CLC1725 (MONT), both C. Cripps. Summit County, Herman Gulch Trailhead, 3200 m, with *Salix* spp., 26 Aug 1983, DBG-F-005617, V.S. Evenson. MONTANA: Carbon County, Beartooth Plateau, site 1, 3000 m, in pure *D.octopetala*, 27 July 2004, CLC1980 (MONT), C. Cripps; N of East Summit, with *Dryas* and *Salixreticulata*, 30 July 1997, ZT6100 (ETH), E. Horak.

##### Discussion.

Grilli and co-workers (2016) showed that in ITS ML analyses *H.velutipes* falls into three unsupported clusters, i.e. one with *H.incarnatulum*, one with *H.leucosarx*, and one with *H.subconcolor*. The latter is discussed above; the former two species do not occur in the kind of habitats sampled in the Rocky Mountains (Beker et al. 2016; Grilli et al. 2016). *Hebelomavelutipes* cannot be distinguished from these three species based on ITS, but it is distinct from all other species treated in Beker et al. (2016). The reason for the intraspecific variation observed in *H.velutipes* has already been shown by Aanen et al. (2001), namely that *H.velutipes* possesses ITS alleles that differ greatly. In the Rocky Mountains, representatives of two of the clusters were found, the *H.leucosarx* cluster and the *H.subconcolor* cluster, and the collections from Montana fall into the first of these clusters while those from Colorado fall in the latter cluster. Accordingly, the number of differences are between 2–23 [0–5] bp; seven pairs with 2–6 [0–1] bp differences and seven pairs with 20–23 [2–5] bp differences. Looking at all included collections, the overall figure hardly changes (1–23 bp), although the collections randomly selected from the FE dataset include representatives of all three clusters (Fig. 5). To date we have not observed any morphological or ecological differences between members of the different clusters. The geographical differentiation of the RM representatives of *H.velutipes* is possibly a sampling artifact.

This species displays the characteristic features of *H.* sect. *Velutipes*, i.e. the absence of a veil, presence of a velutinate stipe, and rather strongly dextrinoid spores (reaction can take a while), as well as the gently clavate cheilocystidia. It is known to be common and widely distributed in Europe at lower elevations primarily with deciduous trees but also with coniferous hosts. There are a number of arctic and alpine records, particularly from Svalbard with *Dryasoctopetala* and *Salixpolaris* (Beker et al. 2016), and it has been previously reported from the North American alpine zone (Beker et al. 2010). This species produces relatively large basidiomes for the genus in the alpine; but because of its pale coloration and lack of a veil, young specimens may have been incorrectly identified as *H.alpinum* or *H.hiemale*, which are typically smaller. Phylogenetically *H.velutipes* is not close to these two species but, as mentioned, is related to *H.subconcolor*, which is smaller with fewer lamellae, grayer coloration and is also reported from the Rocky Mountain alpine zone. Interestingly, almost all Rocky Mountain specimens of *H.velutipes* were found with *Dryas*, which might help with field recognition, in addition to its robust stature, and stout white stipe.

#### 7. Hebeloma alpinum

Taxon classificationFungiAgaricalesHymenogastraceae

(J. Favre) Bruchet, Bull. Mens. Soc. Linn. Lyon 39 (6 suppl.): 68 (1970)

**Figure 13.**
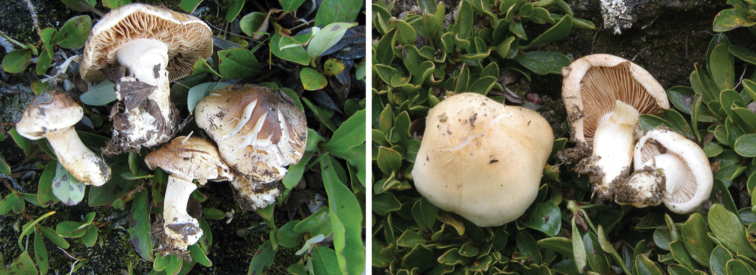
*Hebelomaalpinum*, CLC2855 and HJB11123 (Switzerland).

Figures 4A , 13 , 23 (7)

##### Etymology.

*alpinum* from the alpine.

##### Description.

Cortina absent. Pileus 20–35 mm in diameter, convex to broadly domed, buff to pale brown, rarely brown, slightly paler at margin but not two-toned, smooth, cracking when dry; margin turned down or in. Lamellae attached, emarginate, somewhat broad, pale milk coffee, L = 40–70 plus lamellulae; edges white fimbriate, beaded. Stipe 15–30 × 4–10 mm, rather short, equal, sometimes slightly restricted in middle, clavate, white, firm. Context buff. Odor slightly raphanoid. Exsiccate: pileus brown, slightly caramel color; lamellae dark rusty brown; stipe short, cream color.

Basidiospores yellowish brown, amygdaliform with a snout, more symmetrical in side view, apiculate, sometimes guttulate, weakly ornamented (O1, O2), no loosening perispore noted (P0), very slightly dextrinoid (D0, D1), 10–12 × 6–7 µm, on average 11.2 × 6.6 µm, a few large spores present –18 × –8 µm, Q = 1.69. Basidia 32–40 × 8.5–10.5, mainly four-spored, some possibly two-spored. Cheilocystidia mostly clavate-stiptate, 55–75 µm long, apex width 6.5–10.5 µm, median width 4–5.5 µm, base width 3.5–4.5 µm. Pleurocystidia absent. Epicutis thickness 60–160 µm, with some encrusted hyphae.

##### Rocky Mountain ecology.

Information is based on one collection from Montana, with mixed dwarf and shrub *Salix* species.

##### Rocky Mountain specimens examined.

U.S.A. MONTANA: Park County, Lulu Pass, 3000 m in *Salixarctica* and *S.glauca*, 11 Aug 2012, CLC2855 (MONT), C. Cripps.

##### Discussion.

The only confirmed report we have for this species from the Rocky Mountains relies on a single collection of a few specimens found near Cooke City, Montana at an elevation of 3000 m with dwarf and shrub *Salix* species. In the network Fig. 4A, this single RM representative of *H.alpinum* appears rather distant from its European counterparts, which are clustered at one of the centers of the network, i.e. the biggest circle, of the *H.alpinum* complex. An ITS tree including the *H.alpinum* complex is given in Eberhardt et al. (2015a). Although this collection appears molecularly quite far removed from its conspecifics, 6–10 [1–2] bp, the total distance is largely due to a 5 bp indel repeating a sequence motif generally present in members of the *H.alpinum* complex. Thus, the molecular results do not argue against this being *H.alpinum*. This species is quite variable molecularly as well as morphologically (see the discussion of the alpinum-complex in Beker et al. 2016). The spores of this collection are on the lower end of the range for this taxon, as given in Beker et al. 2016, but still comfortably within the range.

*Hebelomaalpinum* has been reported previously in North America from the Rocky Mountain alpine zone (Cripps and Horak 2008) and Alaska (Miller 1998), however, most sightings were not molecularly confirmed. There are three records from the Canadian Arctic collected in 1971 and 1974 (Ohenoja and Ohenoja 2010), which have been confirmed molecularly (Beker et al. 2018). Ten collections at the Denver Botanic Garden, originally labeled *H.alpinum*, are now molecularly confirmed as *H.hiemale* (see comments for this species).

Favre originally described this species from the Swiss Alps as Hebelomacrustuliniformevar.alpinum Favre (Favre 1955) and Bruchet (1970) elevated it to species level. *Hebelomaalpinum* appears confined to arctic-alpine habitats and has been reported from such regions of the European Alps, Carpathians, Pyrenees, Greenland, Iceland, Scandinavia, Svalbard, and Switzerland, primarily with *Salixreticulata*, *S.polaris*, *S.retusa*, and *Dryasoctopetala* as well as *Persicaria* (Beker et al. 2016). The species is in H.sect.Denudata, subsect. Crustuliniformia because of the lack of a veil, the clavate-stipitate shape of the cheilocystidia and molecular data (Eberhardt et al. 2015a). As a relatively robust alpine species, it should be compared to *H.hiemale* and *H.velutipes*; the latter has a robust floccose white stipe.

### Hebeloma section Hebeloma

We will address this next section in two parts, again following the outline of the key: first those that have ellipsoid indextrinoid spores (*H.alpinicola*, *H.dunense*, *H.excedens*, *H.marginatulum*, and *H.mesophaeum*), also referred to as the *H.mesophaeum* complex and secondly those with amagdaliform spores that are rather strongly dextrinoid (*H.hygrophilum*, *H.nigellum*, *H.oreophilum*, and *H.spetsbergense*), also referred to as the *H.nigellum* complex.

### HebelomasectionHebeloma, Part one: cortina present, spores ellipsoid, not dextrinoid

#### 8. Hebeloma marginatulum

Taxon classificationFungiAgaricalesHymenogastraceae

(J. Favre) Bruchet, Bull. Mens. Soc. Linn. Lyon 39 (6, suppl.): 43 (1970)

**Figure 14.**
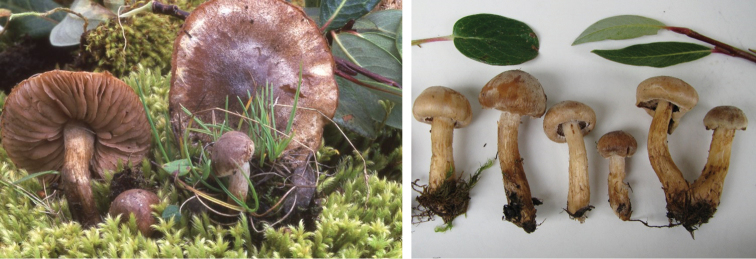
*Hebelomamarginatulum* DBG-F-020841 and CLC3545.

Figures 6B , 14 , 23 (8)

##### Etymology.

From *marginatus*, with a margin or border, emphasizing a thin line of tissue near the margin.

##### Description.

Cortina present, remnants distinctly present in some. Pileus 15–40(–50) mm in diameter, slightly conic-convex, domed convex, irregular, sometimes with a flat center that can even be dished, smooth or rough due to velipellus, shiny, strongly canescent, underneath dark brown, dark chestnut, to dark caramel color, mostly uniform but two-toned in some and then lighter at margin (more hoary, dingy whitish, or ochraceous in one), with a fine white border around the pileus perimeter a few mm in from margin, not hygrophanous; margin turned down or in, rather persistently so, and then covered with copious veil, often irregular, wavy, fragile. In one collection, the cuticle is rather thick and rubbery. Lamellae deeply emarginate and squared off, some pulling away, somewhat broad, L = 30–40 plus lamellulae, cream, then pinkish buff, darkening to medium coffee brown; edges fimbriate. Stipe 20–40(–45) mm × 2–6(–10) mm, equal, undulating or not, pale buff (some with possible yellow tint), and dark (up to black) at base, pruinose at apex, longitudinally fibrous lower, with a few longitudinal fibrils. Context dingy whitish, some with yellowish tones and dark at base. Odor raphanoid or sourish, sometimes faint. Exsiccate: pileus pale brown to dark brown, some obviously canescent; lamellae medium brown; stipe buff or ocher, darker at base.

Basidiospores yellowish gray, pale in Melzers, elliptical with rounded end, inequilateral in side view, no big apiculus, not guttulate, smooth to slightly punctate or rough (O1, O2), indextrinoid (D0, D1), perispore not loosening (P0), 9–12(–13) × 5.5–7(–8) µm, on average 10.1 × 6.4 µm, Q = 1.59. Basidia 25–35 × 8–9 µm, clavate, two and four-spored. Cheilocystidia lageniform, ventricose, often with very long equal neck, and somewhat gradually swollen base, occasionally clavate at apex, sometimes cylindrical, 35–80 µm long × 4–7 µm at apex, 4–6 in middle, and 7–12 (13) at base, no thickening noticed. Pleurocystidia absent. Epicutis thickness 40–100 µm, with some encrusted hyphae.

##### Rocky Mountain ecology.

In the Rocky Mountain alpine zone, with various willows, including dwarf willows *Salixarctica* and *S.reticulata*, and *shrub willow S.planifolia.* Known from both Colorado and Montana.

##### Rocky Mountain specimens examined.

U.S.A. COLORADO: Front Range, Loveland Pass, 12 Aug 2013, in *Dryas*, DBG-F-027694, C. Cripps; 12 Aug 2013, with *Salix* sp., DBG-F-027695, C. Cripps; 25 Aug 2000, with *Salix* sp., DBG-F-020708, V.S. Evenson; 21 Aug 2003, with *Salix* sp., DBG-F-021388, V.S. Evenson; 20 Aug 2013, DBG-F-027682, L. Gillman; 21 Aug 2003, with *Salix* sp., DBG-F-021405, O.K. Miller, Jr; San Juan County, Cinnamon Pass, 3700 m, with *Salixarctica*, 29 July 2000, CLC 1413 (MONT), C. Cripps, 3700 m, with *Salixarctica*, 27 July 2002, CLC1811 (MONT), C. Cripps; 29 July 2000, with *S.reticulata* and *Salix* sp., ZT9002 (ETH), E. Horak; Black Bear Basin, 2 Aug 2000, 3830 m, with *S.planifolia*, CLC1448 (MONT), C. Cripps; 8 Aug 2000, with *S.arctica*, CLC1449 (MONT), C. Cripps; 11 Aug 2001, with *S.reticulata*, ZT9813 (ETH), E. Horak; 3760 m, with *Salixarctica*, 11 Aug 2001, CLC1718 (MONT), C. Cripps; Emma Lake/Horseshoe Basin, 3688 m, with *S.arctica*, 31 July 2002, CLC1874 (MONT), C. Cripps; 31 July 2002, with *S.arctica*, CLC1880 (MONT), C. Cripps; Imogene Pass, 29 July 2002, 3850 m, with *S.arctica*, CLC1836 (MONT), C. Cripps; Mineral Basin, 3850 m, with *S.arctica*, 29 July 2002, with *S.arctica*, CLC1840 (MONT), C. Cripps; without obvious host, although *Salix* in the vicinity, 30 July 2002, CLC1860 (MONT), C. Cripps; with *S.arctica* and *S.planifolia*, 30 July 2002, CLC1861 (MONT), C. Cripps; 3835 m, with *S.arctica*, 7 Aug 2001, CLC1667 (MONT), C. Cripps; Stony Pass, 3840 m, with *S.arctica*, 28 July 2002, CLC1824 (MONT), C. Cripps; 3840 m, with *S.arctica*, 28 July 2002, CLC1826 (MONT), C. Cripps. Sawatch Range, Independence, 3 Aug 2000, with *Salix* sp., DBG-F-020841, DBG-F-020856, V.S. Evenson; 3 Aug 2000 with *Salix* sp., DBG-F-020843, V.S. Evenson; 3760 m, with *S.planifolia*, 7 Aug 2000, CLC1478 (MONT), C. Cripps. MONTANA: Carbon County, Beartooth Plateau, site 1, 9 Sept 2000, with *S.planifolia*, CLC1545 (MONT), C. Cripps; Quad Creek, 8 Aug 2008, with *S.planifolia*, HJB12458, A. and M. Ronikier; 11 Aug 2017; with *Salixreticulata* and *S.planifolia*, 11 Aug 2017, CLC3545 (MONT), C. Cripps.

##### Discussion.

*Hebelomamarginatulum* is distinct from other species of the *H.mesophaeum* complex, but not by much as to molecular distance (Fig. 6B). The species is paraphyletic in relation to the monophylum including the other taxa of the complex. With 0–19 [0–2] bp, the intraspecific variation is quite extensive in *H.marginatulum* in terms of total differences. Within each dataset, the ITS variation is also quite large, 0–14 [0–2] bp for the RM (29 sequences) and 0–17 [0–2] bp for the FE dataset (21 sequences). However, the total number of considered sequences is also larger than for other species.

This taxon was first described as H.versipellevar.marginatulum by Favre (1955) from the alpine region of the Swiss Alps and was later raised to species level by Bruchet (1970). It is now considered to be restricted to arctic and alpine habitats primarily with dwarf willows (Beker et al. 2016, 2018). Confirmed records show it to be present in these habitats in Canada, Greenland, Iceland, Scandinavia, Svalbard as well as the European Alps and the Carpathians and Rocky Mountains (Eberhardt et al. 2015b; Beker et al. 2016). Vesterholt (2005) described *H.polare* as a darker brown closely related species, but this has been synonymized with *H.marginatulum* (Beker et al. 2016). The Rocky Mountain specimens are also mostly uniformly dark brown with a canescent sheen.

Collections from the alpine that are very hoary and dark brown have been misinterpreted as *H.bruchetii* Bon (Miller and Evenson 2001) before molecular techniques; *H.bruchetii*, first described as an alpine species, has now been synonymized with *H.mesophaeum* and should have smaller spores. *Hebelomamarginatulum* is mentioned as a subalpine species (in Idaho) by Smith et al. (1983) who described two varieties (ver. fallax, var. proximum) from the subalpine in Colorado. Smith’s spore descriptions (dextrinoid with sharp ends) for his varieties may not fit this species, but the authors recognize that these varieties of *H.marginatulum*, and indeed other closely related species, need more study in North America.

This species is in H.sect.Hebeloma because of basidiomes with a cortina and the ventricose cheilocystidia together with the non-dextrinoid, or barely dextrinoid, spores that are primarily elliptical; within this group, it has an arctic-alpine habitat and relatively large spores (greater than 10 × 6 µm).

#### 9. Hebeloma alpinicola

Taxon classificationFungiAgaricalesHymenogastraceae

A.H. Sm., V.S. Evenson & Mitchel, Veiled species of Hebeloma in the western United States (Ann Arbor): 48 (1983)

**Figure 15.**
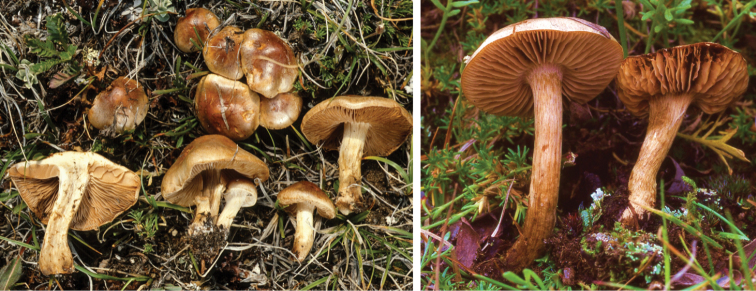
*Hebelomaalpinicola*, DBG-F-020565 and CLC1577.

Figures 6B , 15 , 23 (9)

##### Etymology.

*alpini*- and *cola*, meaning dweller, to emphasise its alpine habitat, although this taxon is not found exclusively in such habitats.

##### Description.

Cortina present. Pileus robust, fleshy, 20–40 mm in diameter, irregular convex, somewhat domed or not, reddish brown center with grayish tones, outwards ocher and lighter towards margin (buff not white), not particularly two-toned, with hoary canescent coating that dries shiny; margin turned in at first, and then turned down. Lamellae narrowly attached, slight emarginate, or with a tooth, or pulling away, somewhat broad, milk coffee, L = 36–44; edges white floccose. Stipe 30–40 × 5–10 mm, equal, straight or not, whitish and pruinose at apex, dingy ocher and longitudinally fibrillose and striate in lower part, base sometimes encased in sand or earth. Context dingy whitish, darker below, and flesh staining brown; stipe solid or slightly hollow. Odor raphanoid. Exsiccate: pileus and stipe medium ochraceous brown; lamellae dark brown; stipe base encased in soil in the large collection (CLC1577).

Basidiospores elliptical, or some slightly amygdaliform or ovoid, with rounded end, smooth to slightly rough (O0, O1), small apiculus, not guttulate, not dextrinoid (D0), perispore not loosening (P0), 8–11 × 5–6, on average 9.1 × 5.6 µm, Q = 1.63 Basidia clavate, four-spored, 30–35 × 7–8 µm. Pleurocystidia usually absent but occasionally present, sometimes rostrate. Cheilocystidia mostly cylindrical for the top two thirds and then swollen near the base (lageniform or ventricose), 30–70 µm long × 3–8 µm at apex, 3–7 µm in middle, and 6–11 µm at base, no yellow contents noted. Epicutis thickness up to 200 µm, with no encrusted hyphae recorded.

##### Rocky Mountain ecology.

Collected from two different sites, one in Montana, the second in Colorado. The first site is a mixture of *Dryas*, *Salixplanifolia* and *S.reticulata*, with some *Persicaria* present. The second site is a low alpine zone with dwarf willows. In both cases the growth habit was gregarious, sometimes in rings, sometimes cespitose, but not completely joined.

##### Rocky Mountain specimens examined.

U.S.A. COLORADO: Gilpin County, Roosevelt National Forest, Little Echo Lake shoreline, near dwarf willows, 3500 m, 4 Sept 1999, DBG-F-020565, V.S. Evenson, M. Brown; 4 Sept 1999, DBG-F-020582, V.E. Evenson. MONTANA/WYOMING state line: Beartooth Plateau, 3020 m, with *Persicaria*, *Geum*, sedges, grasses, and quite distant *S.planifolia*, 19 July 2001, CLC1577 (MONT), C. Cripps; Quad Creek, 4 Aug 2008 with *Dryasoctopetala* and *S.reticulata*, HJB12439, C. Cripps.

##### Other specimens examined.

See Table 2.

##### Discussion.

Figure 6B shows *H.alpinicola* as paraphyletic and closely related but not mixed with species from the *H.mesophaeum* complex other than *H.marginatulum*. The *H.alpinicola* representatives differ by 0–13 [0–2] bp from each other. Based on morphology and ITS results, the types of seven species, namely *H.alpinicola*, *H.chapmaniae* A.H. Sm., *H.littenii* A.H. Sm., *H.nigromaculatum* A.H. Sm., *H.perigoense* A.H. Sm., *H.smithii* = *H.angustifolium* A.H. Sm. et al. nom. illegit. (the name *Hebelomaangustifolium* (Britzelm.)Sacc. already existed) and *H.subargillaceum* A.H. Sm. are synonyms. The inclusion of the seven types increases the absolute intraspecific variation to 0–16 [0–4] bp. The distance from other species of the complex is 3–22 [0–7] bp within the sample. Although *H.alpinicola* has not yet been fully tested in multilocus analyses, we consider its distinctive morphology combined with the ITS evidence to be sufficient to assign the four RM collections to this species.

This taxon, with its small ellipsoid, indextrinoid spores and ventricose cheilocystidia is a member of H.sect.Hebeloma. Morphologically it is closely related to *H.excedens* and *H.mesophaeum.* It is generally more robust than these two species, especially the stipe, and the pileus is not as two-toned. Colorado collections were described as having gray tones. While further work is needed to decide whether this really is a species distinct from the other two, the molecular evidence coupled with the morphological evidence suggest this to be the case. We have studied a number of collections, from a variety of habitats within North America that all appear to represent this taxon. *Hebelomachapmaniae*, *H.littenii*, *H.nigromaculatum*, *H.perigoense*, and *H.subargillaceum* were all published by Smith et al. (1983) in the same publication that featured *H.alpinicola*; the replacement name *H.smithii* is later (Quadraccia 1987). Although there is some molecular variation between these seven collections, it is very small and we see insufficient evidence to separate these species. We have selected the name *Hebelomaalpinicola* on the grounds that although not all collections are strictly alpine, the majority are at least subalpine.

#### 10. Hebeloma dunense

Taxon classificationFungiAgaricalesHymenogastraceae

L. Corb. & R. Heim, Mém. Soc. Natn. Sci. Nat. Math. Cherbourg 40: 16 (1929)

**Figure 16.**
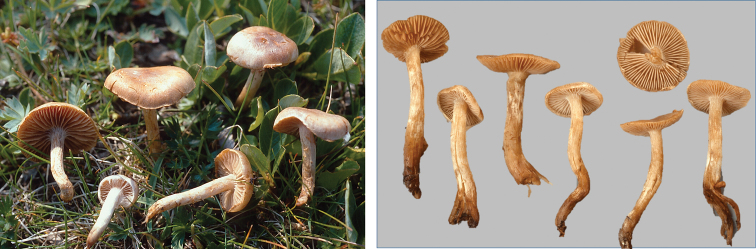
*Hebelomadunense*, CLC1821 and CLC1845.

Figures 6B , 16 , 23 (10)

##### Etymology.

Originally found in sand in dunes.

##### Description.

Cortina present. Pileus 10–28 mm in diameter, convex, slightly conic-convex, with or without a slight umbo (one papillate), or almost applanate, some sunken in center, smooth, greasy, pale pinkish buff at first, becoming caramel color in center, outwards remaining pale, with a hoary coating, some flecks of white in outer part, mostly appearing pale unicolor; margin turned in or down, covered with white veil tissue or not. Lamellae emarginate to subdecurrent, or pulling away, variable, L = 25–48 plus lamellulae, a bit distant, cream buff to pinkish buff at first, then milk coffee; edges white fimbriate. Stipe 20–50 × 2–6 mm, equal or narrowing a bit at base, dingy whitish buff in top part, sometimes pruinose and base darkening to golden color then blackish brown (not always obvious unless cut open), with fibrils on lower part and/or a few ‘patches of tissue’. Context dingy white, watery buff, dark at base, sometimes splitting, often hollow when mature; tough in base. Odor faintly raphanoid or absent. Exsiccate: mostly pale; pileus buff or more ochraceous buff, center a bit caramel or not; lamellae pale light ocher; stipe buff, not obviously darker at base.

Basidiospores yellowish gray in Melzer’s, mostly elliptical, a few slightly amygdaliform but typically without much snout, no big apiculus, not guttulate, look smooth but may be slightly rough in Melzer’s (O1, O2), not or only very slightly dextrinoid (D0, D1), and no perispore loosening (P0), 9.5–11.5 × 5.5–7 µm, on average 10.3 × 6.2 µm, Q = 1.65. Basidia 20–30 × 8–9 µm, clavate, four-spored mostly. Pleurocystidia absent. Cheilocystidia cylindrical in the upper part and slightly swollen to more swollen at the base, 40–55 µm long × 4.5–6 µm at apex, 4–6 µm in middle, and 7–10.5 µm wide at base, with occasional thickening of the apical wall, some septate and clamped; many with dense yellow contents. Epicutis thickness 25–75 µm, with some encrusted hyphae.

##### Rocky Mountain ecology.

In the alpine zone of the San Juan Mountains, with dwarf willows *S.reticulata* and *S.arctica*, and shrub willow *S.planifolia*, some in moss or near streams.

##### Rocky Mountain specimens examined.

U.S.A. COLORADO: San Juan County, San Juan Mountains, Cinnamon Pass, 3700 m, with dwarf *Salix* near stream, 29 July 2000, CLC1411 (MONT), C. Cripps; with *Salixreticulata*, 8 Aug 2000, CLC1434 (MONT), C. Cripps; 29 July 2000 with *Salixreticulata*, ZT9001 (ETH), E. Horak; Stony Pass, 3840 m, with *S.arctica*, 28 July 2002, CLC1821 (MONT), C. Cripps; Mineral Basin, with *S.arctica* and *S.planifolia*, in moss, 3835 m, 30 July 2002, CLC1845 (MONT), C. Cripps.

##### Discussion.

Based on ITS data, *Hebelomadunense* is phylogenetically not clearly distinguishable, but neither is it molecularly identical to other members of the *H.mesophaeum* complex (Fig. 6B). The intraspecific variation is 0–10 [0–2] bp (17 sequences), within the RM dataset (5 sequences), 1–7 [0–1] bp. The exclusively RM circle in Fig. 6B is a result of the data selection; this corresponds to ITS variants that do occur in the FE dataset, but did not come up in the random selection of sequences for this species.

For the Rocky Mountain collections, so far, *H.dunense* has been found more often with dwarf willows *S.arctica*, *S.reticulata*, and shrub willow *S.planifolia* in contrast to *H.mesophaeum* and *H.excedens*, which were more often with *S.glauca*. Originally described from low-elevation dunes with *Salix*, this species has been more recently recognized in arctic and alpine habitats and from Canada, Greenland, Svalbard, the European Alps, and the Carpathians (Beker et al. 2016; Beker et al. 2018; Eberhardt et al. 2015b).

Rocky Mountain specimens of *H.dunense* are pale, often with narrow subdecurrent lamellae; the cortina can be scant or absent, some cheilocystidia have dense yellow contents, and the spores, which are ellipsoid and distinctly but not strongly ornamented, are slightly larger than those of *H.mesophaeum* and *H.excedens*.

#### 11. Hebeloma mesophaeum

Taxon classificationFungiAgaricalesHymenogastraceae

(Pers.) Quél., Mém. Soc. Émul. Montbéliard, sér. 2, 5: 128 (1872)

**Figure 17.**
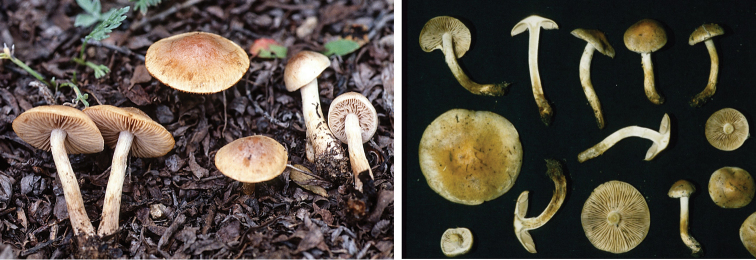
*Hebelomamesophaeum*, CLC1245 and ZT8082.

Figures 6B , 17 , 23 (11)

##### Etymology.

From Greek *meso*, in the middle, and *phaeus*, dark-colored. Persoon (1872) particularly mentioned the peculiar reddish brown pileus center “disco rufo-fusco peculiaris” which is characteristic of this taxon.

##### Description.

Cortina present. Pileus 10–20 mm in diameter, convex with low indistinct umbo, or conic-convex, smooth, shiny, greasy, yellowish brown in center, outwards lightening to pale ocher, at margin buff, two-toned, non-translucent; margin entire, turned in when young, covered with veil or not. Lamellae attached, adnate, L = 38–40, pale buff, pinkish buff, then pinkish brown; edges fimbriate. Stipe: 30–45 × 3–5(–8 at base), very gradually larger at base, white, pruinose at apex, and fibrillose and darker below to ocher yellow and then blackish at very base. Context pale, dark in base of stipe. Odor raphanoid. Exsiccate: pileus pale brown, stipe with yellow sheen and darker at base.

Basidiospores yellow brown, elliptical, a few slightly ovoid, no big apiculus, not guttulate, looks almost smooth even under high magnification (O1), not or only very slightly dextrinoid (D0, D1), and no perispore loosening (P0), 8–10.5(–11) × 5–6.5 µm, on average 9.7 × 5.8 µm, Q = 1.66. Basidia 20–30 × 6–9 µm, clavate, four-spored mostly. Pleurocystidia absent. Cheilocystidia cylindrical in the upper part and slightly swollen to more swollen at the base, rarely fully cylindrical, 30–55 µm long × 4–7 µm at apex, 4–7 µm in middle, and 6–9.5(–10.5) µm wide at base, with occasional thickening of the apical wall, some septate. Epicutis thickness 60–350 µm, with some encrusted hyphae.

##### Rocky Mountain ecology.

Known so far only from the Colorado alpine with *Salixglauca.*

##### Rocky Mountain specimens examined.

U.S.A. COLORADO: Sawatch Range, Independence Pass, 3760 m, with *Salixglauca*, 8 Aug 1998, CLC1245 (MONT), C. Cripps; Front Range, Loveland Pass, 7 Aug 1999 with *Salix* sp., ZT8082 (ETH), E. Horak.

##### Discussion.

Only two collections from the RM dataset turned out to be *H.mesophaeum* that differ in their ITS region by 7 [2] bp (Fig. 6B). The sequence variation among all *H.mesophaeum* sequences (12) of the sample is 1–11 [0–4] bp. Beker et al. (2016) did not manage to delimit *H.mesophaeum* based on several loci. They suspected that there might be several species hidden within the sample assigned to *H.mesophaeum*. It appears likely that *H.excedens* and *H.alpinicola* are among these ‘cryptic’ taxa. We made sure that the 10 selected sequences from the FE dataset belong to *H.mesophaeum* in the strict sense. Among the *H.mesophaeum* representatives of the RM dataset, there is one collection that is reminiscent of *H.pubescens*. However, because of its ambiguous morphology we decided to keep it in *H.mesophaeum*. The respective collection (CLC1245) differs by 2–4 [1–2] bp from the available *H.pubescens* data (3 sequences).

Previously *Hebelomabruchetii* Bon was one of the most commonly reported species from arctic and alpine areas, but it has now been synonymized with and folded into *H.mesophaeum* (Beker et al. 2016). *Hebelomamesophaeum* has relatively small elliptical spores that are smooth to slightly rough and not dextrinoid. *Hebelomamesophaeum* is a widespread species reported in almost all arctic and alpine habitats, as well as from subalpine, boreal, and lower elevation habitats with a wide variety of hosts (Beker et al. 2016). Also, many varieties have been described in North America (Smith et al. 1983) and in Europe (Vesterholt 2005). Some of the European taxa have been synonymized by the authors (Beker et al. 2016) and it remains to check the 12 North American varieties delineated by Smith et al. (1983).

#### 12. Hebeloma excedens

Taxon classificationFungiAgaricalesHymenogastraceae

(Peck) Sacc., Syll. Fung. 5: 806 (1887)

**Figure 18.**
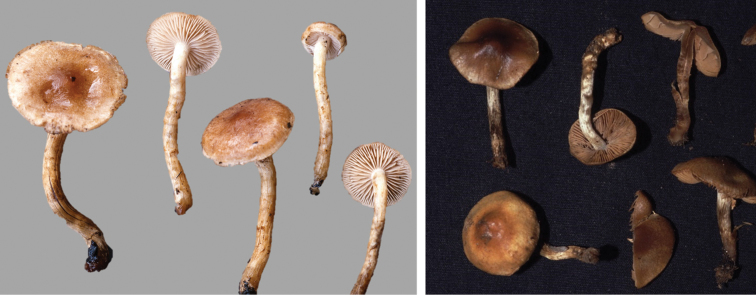
*Hebelomaexcedens*, CLC1685 and ZT9830.

Figures 6B , 18 , 23 (12)

##### Etymology.

For the pileus cuticle which can exceed the lamellae.

##### Description.

Cortina present. Pileus 10–25 mm in diameter, shallow convex, campanulate, then almost applanate, slight umbo or not, viscid or greasy, medium cocoa brown to orange caramel in center and pale brown on most of the pileus, with or without white tissue at margin, or with whitish rim; margin originally described as extending beyond the lamellae. Pileus thin-fleshed. Lamellae sinuate, subdecurrent, narrow, becoming broader and eroded, very pale, cream with pinkish buff tint, L = 32–48 plus lamellulae. Stipe 30–50 × 2–4 mm, equal, slightly curved, pale cream, silky, pruinose above ring zone, more dingy brown below but still pale, with a golden brown fibrils in zones, blackening towards base. Context whitish in pileus and stipe apex and yellowish brown in lower stipe down to blackish at base; stipe tough, rubbery. Odor: raphanoid or none. Exsiccate: small, pale buff overall, base of stipe dark in some.

Basidiospores yellow brown, elliptical, a few slightly ovoid, no big apiculus, not guttulate, looks almost smooth to very slightly rough even under high magnification (O1), not or only very slightly dextrinoid (D0,D1), and no perispore loosening (P0), 7–11 × 5–6.5 µm, on average 9.1 × 5.8 µm, Q = 1.55. Basidia 20–30 × 6–9 µm, clavate, four-spored mostly. Pleurocystidia absent. Cheilocystidia cylindrical in the upper part and slightly swollen to more swollen at the base, rarely fully cylindrical, 30–60 µm long × 4–7 µm at apex, 4–6.5 µm in middle, and 6–10 µm wide at base, some septate. Epicutis thickness 65–200 µm, with some encrusted hyphae.

##### Rocky Mountain ecology.

In alpine with shrub willow *Salixglauca*, Colorado.

##### Rocky Mountain specimens examined.

U.S.A. COLORADO: San Juan County, San Juan Mountains. U.S. Basin, 3658 m, with *Salixglauca*, 8 Aug 2001, CLC1685 (MONT), C. Cripps. Sawatch Range, Independence Pass, 14 Aug 1999 with *Salix* sp., ZT7475 (ETH), E. Horak; 12 Aug 1999 with *Salix* sp., ZT8136 (ETH), E. Horak; 14 Aug 2001 with *Salixglauca* and *S.planifolia*, ZT9830 (ETH), E. Horak; 3760 m, with *Salixglauca*, 13 Aug 2001, CLC1732 (MONT), C. Cripps. Front Range, Loveland Pass, 7 Aug 1999 with *Salix* sp., ZT8074 (ETH), E. Horak.

##### Other specimens examined.

NEW YORK: Saratoga at approx. 100 m, with *Pinus* sp. on sandy soil in woodland, Oct 1870, NYS-F-001123, C.H. Peck (**holotype**).

##### Discussion.

*Hebelomaexcedens* was not treated by Beker et al. (2016). The type of *H.excedens* fits in with the majority of the RM *H.excedens* collections, but the species cannot be clearly separated from *H.mesophaeum* (Fig. 6B). Looking at absolute differences, the intraspecific variation of the *H.excedens* sample (RM + type = 7 sequences) is 0–8 [0–1] bp, whereas the variation in the sample between *H.excedens* and *H.mesophaeum* is 2–11 [0–4] bp. In terms of absolute differences, the type of *H.excedens* is 5–8 [0–1] bp different from other collections referred to this species, but as Fig. 6B shows it is not strongly differentiated from other members of *H.excedens*, if ambiguous positions are treated as missing data as in networks or equated to their constituting bases as in the ML tree. In terms of absolute differences, the type of *H.excedens* is 5–11 [0–3] bp away from the *H.mesophaeum* sequences of the sample. Thus, within the limited support ITS data can give in this case, we do consider the species identification of the RM *H.excedens* collections as molecularly supported. Until the question of the distinctness and delimitation of this species can be clarified, we prefer to treat it as an independent taxon.

*Hebelomapubescens* Beker & U. Eberh. is another species from the *H.mesophaeum* complex that might occur in the sampled habitats of the Rocky Mountains and is close to *H.excedens* in Fig. 6B. Based on a small sample (3 sequences available for *H.pubescens*; 7 sequences for *H.excedens*), the species vary 5–10 [1–3] bp in their ITS region.

*Hebelomaexcedens* was first described by North American mycologist C.H. Peck; the species, with its lageniform to ventricose cheilocystidia and small elliptical, almost smooth, indextrinoid spores belongs to H.sect.Hebeloma. It is closely allied with *Hebelomamesophaeum*, with which we believe it has often been confused. Separating these two taxa morphologically is rather difficult, but it does appear that the pileus of *H.excedens* may be more evenly colored, less yellow brown, less brown in the center, and it was originally described as having a cuticle that extended beyond the lamellae. The stipe surface appears to have fibrils arranged in zones, in contrast to that of *H.mesophaeum*. However, further work is required before we can have confidence that these characters are consistently different.

We have examined a number of collections from North America that are morphologically and molecularly consistent with this taxon. Based on these studies it would appear that *Hebelomaexcedens* is widespread across North America and occurs in a wide variety of habitats.

### HebelomasectionHebeloma, Part two: cortina present, spores amygdaliform, rather strongly dextrinoid

#### 13. Hebeloma oreophilum

Taxon classificationFungiAgaricalesHymenogastraceae

Beker & U. Eberh., Mycologia 107: 1295 (2016) [2015]

**Figure 19.**
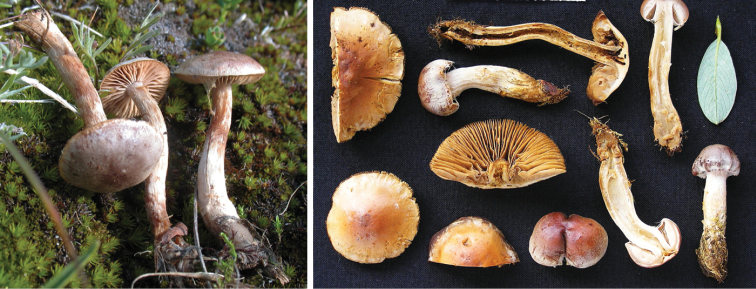
*Hebelomaoreophilum*, DBG-F-027674 and ZT12733.

Figures 6A , 19 , 23 (13)

##### Etymology.

From *oreophilus*, mountain loving to emphasize its presence in alpine habitats.

##### Description.

Cortina present. Pileus 15–30 mm in diameter, convex, hemispherical, not umbonate, smooth, dry or greasy, medium brown, bay brown, reddish brown, dark black brown, with white to cream rim of fibrillose veil remnants at margin, with hoary coating; margin even or weakly scalloped. Thick waxy pellicle mentioned in one collection. Lamellae emarginate, subdistant, L = 40–50 plus lamellulae, cream at first then milk coffee color, pinkish cinnamon; margin floccose, white. Stipe 15–60 × 3–8 mm, equal or slightly enlarged at base, a bit curved or undulating, whitich, tan, brown, in top part and darkening to blackish brown at base, pruinose in top half and fibrous below, with patches of fibrils. Context watery buff with yellow tint, and blackish brown in base, stipe hollow. Odor raphanoid. Exsiccate pale brown all over, not dark.

Basidiospores amygdaliform, with a small snout, apiculate, not guttulate, finely verrucose (O1, O2), distinctly dextrinoid (D2, D3), no perispore loosening observed (P0), 10–14 × 6–8 µm, on average 11.7 × 6.9 µm, Q = 1.68. Basidia clavate, 25–35 × 8–10 µm, mostly four-spored. Cheilocystidia lageniform, with subcapitate apex, long neck (sometimes wiggly), with gradually swollen base, sometimes septate, length 30–70 × 4–7 µm at apex, 3–6.5 µm in middle, and up to 13 µm at base, no thickening noticed. Pleurocystidia absent. Epicutis thickness 40–75 µm, with no encrusted hyphae recorded.

##### Rocky Mountain ecology.

In low alpine with *Salix* species in Montana and Colorado.

##### Rocky Mountain specimens examined.

U.S.A. COLORADO: Clear Creek County, Denver Mountain Park, Summit Lake, 3911 m, in *Salixarctica* and *S.glauca*, 20 Aug 2013, DBG-F-027674, L, Gillman; Summit Lake Park, 3912 m, with *Salix* sp., 22 Aug 2012, DBG-F-022788, L. Gillman; Arapaho National Forest, Nature Trail, Mount Goliath, 3658 m, in *Salix* sp, 1 Sept 1999, DBG-F-020558, V.S. Evenson; Pitkin County, White River National Forest, junction of Montezuma Basin and Pearl Pass, in *Salix* sp., 3658 m, 6 Aug 1999, DBG-F-020053, V.S. Evenson. MONTANA: Carbon County, Beartooth Plateau, Frozen Lakes, with dwarf *Salix*, 26 July 1997, 3200 m, CLC1102 (MONT), C. Cripps; site 2, 3100 m, 8 Aug 2002, CLC1937 (MONT), with *Salixplanifolia*, C. Cripps; Billings Fen, in moss near *S.planifolia*, 3048 m, 23 Aug 2017, CLC 3607 (MONT). WYOMING: Beartooth Plateau, Wyoming Creek, with *Salixplanifolia*, 3176 m, 6 Aug 2008, HJB12449, C. Cripps; Beartooth Plateau, Hell-roaring Plateau, near *Salix* sp., 14 Aug 2007, ZT12733 (ETH), E. Horak.

##### Discussion.

*Hebelomaoreophilum* is a member of the *H.nigellum* complex that cannot always be distinguished from *H.nigellum* based on ITS data (Fig. 6A). In terms of differences, the *H.oreophilum* sequences from the sample (9 RM, 10 FE) differ by 0–9 [0–3] bp; 0–8 [0–1] bp within the RM sample. Most similar to *H.oreophilum* is *H.clavulipes*, which in this sample differs by 1–11 [0–3] bp. The two species do not share the same habitats. The differences between species sharing the same habitats (*H.nigellum* and *H.spetsbergense*) are 3–10 [0–5] bp. Morphologically, the easiest way to separate *H.oreophilum* from *H.hygrophilum* and *H.nigellum* is by the number of full length lamellae, always at least 40 for *H.oreophilum* and less than 36 for the others. *Hebelomaclavulipes* is not known from arctic-alpine habitats and has spores with an average width at most 6.6 µm while the average spore width for *H.oreophilum* is on average at least 6.8 µm. *Hebelomaoreophilum* has a persisting cortina and the lageniform/ventricose cheilocystidia of H.sect.Hebeloma.

This species was first described from the western Carpathians (Slovakia) with *Salixreticulata*, *S.retusa*, or *Dryasoctopetala* on calcareous soil (Eberhardt et al. 2015b). It has since been reported from Canada, Greenland, Scandinavia, Svalbard, and the Rocky Mountains (Beker et al. 2016; Beker et al. 2018).

#### 14. Hebeloma hygrophilum

Taxon classificationFungiAgaricalesHymenogastraceae

Poumarat & Corriol, Fungi Europaei 14 (Lomazzo): 138 (2016)

**Figure 20.**
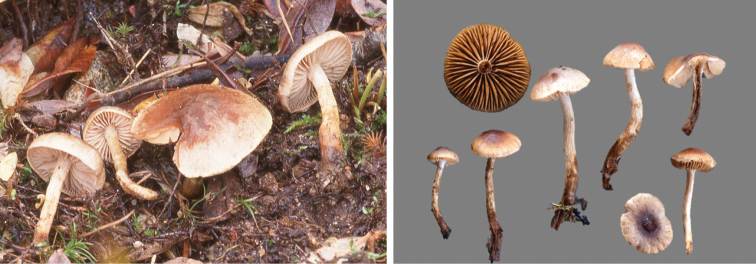
*Hebelomahygrophilum*, DBG-F-021349 and CLC1462.

Figures 6A , 20 , 23 (14)

##### Etymology.

*hygrophilus*, because it is often found in moist, wet, boggy ground.

##### Description.

Cortina present. Pileus 15–25 mm in diameter, convex to almost plane, smooth, greasy, center dark brown, reddish brown, lighter towards margin to buff; margin entire. Lamellae emarginate and strongly curved outwards, a bit distant, L = 24 plus lamellulae, pale buff becoming milk coffee color; edges lighter or darker. Stipe 25–35 × 1–2 mm, long and thin, undulating, dingy cream in top half, darkening to blackish at base, apex pruinose, below with longitudinal fibrils. Context dingy cream and brownish black in stipe base. Odor raphanoid. Exsiccate: small; pileus, two-toned, dark brown center, cream towards margin; stipe thin, whitish with a darker base.

Basidiospores slightly amygdaliform, a few with a snout, apiculate, not guttulate, finely verrucose (O2), distinctly dextrinoid (D2, D3), no perispore loosening observed (P0), 10–13 × 6–7.5 µm, on average 11.4 × 6.8 µm, Q = 1.67; a few spores larger –16 × –7 µm present. Basidia clavate, 25–30 × 7–9 µm, four-spored, possibly some two-spored because of larger spores present. Cheilocystidia lageniform, with subcapitate apex, long neck (sometimes wiggly), occasionally septate, with gradually swollen base, or almost cylindrical, length 35–70 × 4–6.5 µm or wider at apex, 4–6 µm in middle, and up to 7–13 µm at base, no thickening noticed. Pleurocystidia absent. Epicutis thickness 100–130 µm, with some encrusted hyphae.

##### Rocky Mountain ecology.

Based on four collections from Colorado and Montana, in the alpine zone; all with *Salix*, and the presence of *Sphagnum* is mentioned for one.

##### Rocky Mountain specimens examined.

U.S.A. COLORADO: Pitkin/Lake County, Sawatch Range, Independence Pass, 6 Aug 2000, under *S.planifolia*, 3660 m, CLC1462 (MONT), C. Cripps; 7 Aug 2000, *Salixplanifolia*, CLC1476 (MONT), 3660 m, C. Cripps. Summit County, near Summit Lake, with *Sphagnum* sp. and *Salix* sp., 3658 m, 10 Aug 2003, DBG-F-021349, V.S. Evenson. MONTANA: Beartooth Plateau, Frozen Lakes, at 3200 m, near *S.planifolia*, 29 Aug 2002, CLC1948 (MONT), C. Cripps.

##### Discussion.

Figure 6A supports Beker et al. (2016) in that *H.hygrophilum* is paraphyletic in relation to the other members of the *H.mesophaeum* complex based on the ITS sequence, although some genotypes seem to be restricted to this species. The four *H.hygrophilum* representatives from the Rocky Mountains differ by 2–20 [0–2] bp in their ITS, whereas the intraspecific variation of *H.hygrophilum* within the sample is 1–22 [0–3] bp (14 sequences). Responsible for the high distance values is sample CLC1476 (HJB15297), which differs from all other conspecifics by 15–22 [0–1] bp and from all sequences of the ingroup by 14–22 [0–2] bp, while all other *H.hygrophilum* samples differ by only 1–9 [0–2] bp from each other. The morphologically closest taxon occurring in the Rocky Mountains is *H.nigellum* which differs by 3–10 [0–5] (14–21 [0–2]) bp. The values in round brackets are for CLC1476. An unusually high number of SNP positions in CLC1476 is responsible for the large total differences. However, sequences with numerous SNP positions occur occasionally in *Hebeloma* and are normally reproducable (Beker et al. 2016).

*Hebelomahygrophilum* was first described from the Pyrenees in non-alpine habitats above 1250 m (Poumarat and Corriol 2009) and it is known in boreal habitats from northern Europe (Beker et al. 2016). Thus it is typically in subalpine or subarctic habitats. It appears to have been found mostly with *Salix* and usually in wet areas with moss, typically *Sphagnum*. Here we report it for the first time in the alpine habitat (with *S.planifolia*); at least one collection was found in *Sphagnum* moss. It is molecularly close to *H.clavulipes*, *H.nigellum* and *H.oreophilum* (see below). When found in the alpine, it could be confused with *H.nigellum*, which is morphologically very similar. However, the spore width of *H.nigellum* is reported typically with an average over 7 µm, while that for *H.hygrophilum* is reported with an average of less than 7 µm; to add confusion, both appear to have occasional very large spores likely from two-spored basidia.

#### 15. Hebeloma nigellum

Taxon classificationFungiAgaricalesHymenogastraceae

Bruchet, Bull. Mens. Soc. Linn. Lyon 39 (6 suppl.): 126 (1970)

**Figure 21.**
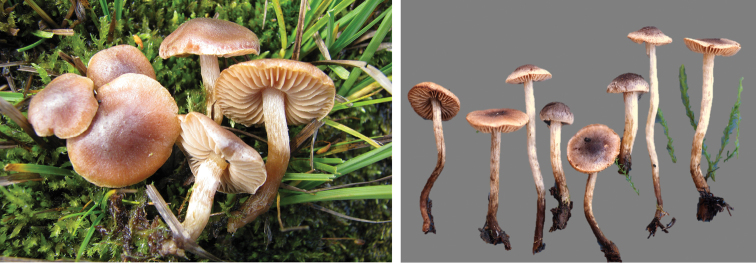
*Hebelomanigellum*, CLC3614b and CLC1420.

Figures 6A , 21 , 23 (15)

##### Etymology.

From *nigellus*, meaning blackish for the dark pileus.

##### Description.

Cortina present. Pileus 8–20 mm in diameter, broadly convex to hemispherical to almost plane with a small umbo, greasy, smooth or slightly fibrous, in center dark date brown, chocolate brown, or blackish brown, at margin paler even to cream, appearing two-toned, with hoary sheen, glazed-looking, not hygrophanous; margin inrolled at first, then even (not rimose). Lamellae emarginate, even with a tooth, normally spaced, L = 24–32 with lamellulae, whitish, then pale milk coffee, pale brown, paleness persisting; edges floccose. Stipe 15–50 × 1.5–4 mm, long and slim, equal, undulating a bit, pale dingy whitish in top half darkening to black brown at base, pruinose at apex, below silky-shiny, smooth to fibrillose. Context dingy whitish, darkening to brownish at base, rubbery in stipe. Odor raphanoid. Exsiccate: pileus small, two-toned, center dark brown, outwards cream; lamellae brown, red-brown; stipe long and very thin, cream, dark at base.

Basidiospores yellowish brown, amygdaliform, a few ellipsoid in certain view, no/slight snout, no big apiculus, slightly rough (O1, O2), perispore occasionally observed loosening very slightly (P0, P1), usually distinctly dextrinoid (D2, D3), not guttulate, 10–14.5 × 6–8 µm, on average 11.9 × 7.2 µm, Q = 1.6. Basidia 27–40 × 7.58–10.5 µm, sterigma 2–3 µm, clavate, mainly four-spored. Cheilocystidia lageniform, more or less swollen at the base, top half cylindrical, some apical thickening, some septate, 30–80 × 3.5–6.5 µm at apex, 3.5–6 µm in middle, 6.5–12.5 µm at base. Pleurocystidia absent. Epicutis thickness 40–75 µm, with no encrusted hyphae recorded.

##### Rocky Mountain ecology.

Alpine mostly near *Salixplanifolia* and in moss; reported from Colorado and Montana.

##### Rocky Mountain specimens examined.

U.S.A. COLORADO: San Juan County, San Juan Mountains, Engineer Pass, in *Salixplanifolia*, 30 July 2000, CLC1420 (MONT), C. Cripps; Cinnamon Pass, in *Salix* spp., 10 Aug 2001, CLC1707 (MONT), C. Cripps. MONTANA: Beartooth Plateau, Frozen Lakes: at 3200 m in moss near *S.planifolia*, 21 Aug 2001, CLC1778 (MONT), C. Cripps; N Pass, with *S.planifolia*, 9 Aug 1998, ZT6425 (ETH), E. Horak; Billings Fen, in moss near *S.planifolia*, 23 Aug 2017, CLC3614b (MONT), C. Cripps.

##### Discussion.

According to Beker et al. (2016), *H.nigellum* is paraphyletic in the ITS region, but monophyletic and bootstrap supported in multi-locus analyses. The corresponding network is in Figure 6A. *Hebelomanigellum* is similar in its variabiltiy within the Rocky Mountains (1–7 [0–1] bp differences based on 5 sequences when compared with the random selection of 11 sequences from the FE dataset (0–8 [0–3] bp). As discussed above, *H.nigellum* is close to and not always distinguishable from *H.hygrophilum* by ITS sequence. Another arctic and alpine species is *H.spetsbergense* (discussed below) that cannot be distinguished from *H.nigellum* by ITS sequence either.

*Hebelomanigellum* is a small, slim species with a dark-centered pileus and rather large, dextrinoid, amygdaliform spores. It is widespread across northern Europe, not only in arctic-alpine habitats, and is reported from alpine and arctic habitats in Canada, Greenland, Iceland, Svalbard and the European Alps (Beker et al. 2016, 2018). In molecular and morphological features it is close to *H.hygrophilum* (which normally associates with *Salix* in non-arctic-alpine habitats). *Hebelomakuehneri* Bruchet, a commonly reported arctic-alpine species, was described in the same paper as *H.nigellum* with the main differentiation being that the former has more brownish coloration and the latter more blackish tones (Bruchet 1970); a distinction that could not be supported by other lines of evidence. The holotype of *H.kuehneri* was lost, however, and a new lectotype (selected from the paratypes) has been established (Beker et al. 2016; LY BR66-15); it is sequenced and is a molecular match to *H.nigellum*. We here follow Beker et al. (2016) in selecting the name *H.nigellum* over *H.kuehneri* for this species.

#### 16. Hebeloma spetsbergense

Taxon classificationFungiAgaricalesHymenogastraceae

Beker & U. Eberh., Fungi Europaei 14 (Lomazzo): 180 (2016)

Figures 6A , 22

##### Etymology.

Originally found in Svalbard.

##### Description.

Cortina present. Pileus 10–25 mm in diameter, shallow convex, almost applanate with indistinct umbo or not, smooth, tacky to dry, brown in center, outwards paler brown or more cinnamon, with white edge, not hygrophanous; margin turned down in young specimens, entire. Lamellae attached, adnexed, medium close, L = 26–30, pale cream to milk coffee, to brown; edges indistinct fimbriate. Stipe long and thin, 20–40 × 2–3 mm, equal, cream at apex to dark brown at base, fibrils at apex, and below silky-smooth with longitundinal fibrils. Context cream and brown to black in lower part. Odor raphanoid. Exsiccata: pileus brown, darker brown in center; lamellae reddish brown; stipe thin, cream but darkening at base.

Basidiospores yellow brown, amygdaliform, without a large snout, apiculate, not guttulate, finely verrucose (O1, O2), distinctly and sometimes strongly dextrinoid (D2, D3), no loosening perispore observed (P0), 11–14 × 7–8.5 µm, on average 12.5 × 7.6 µm, Q = 1.65. Basidia 28–35 × 8–10 µm, clavate, mostly four-spored. Cheilocystidia lageniform, with long cylindrical neck, 30–80 × 4–7 µm at apex, 4–5.5 µm in middle, and 7–10.5 µm at base. Pleurocystidia absent. Epicutis thickness 30–35 µm, with no encrusted hyphae recorded.

**Figure 22.**
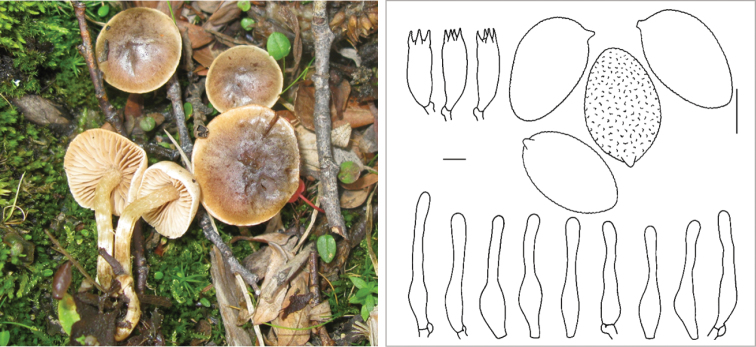
*Hebelomaspetsbergense*, DBG-F-027678 and BR5020184126599 (HJB 11982, from Svalbard). Scale bar for basidia and cheilocystidia 5 µm, for spores 10 µm. Drawing G. Walther, reproduced from Beker et al. (2016).

**Figure 23.**
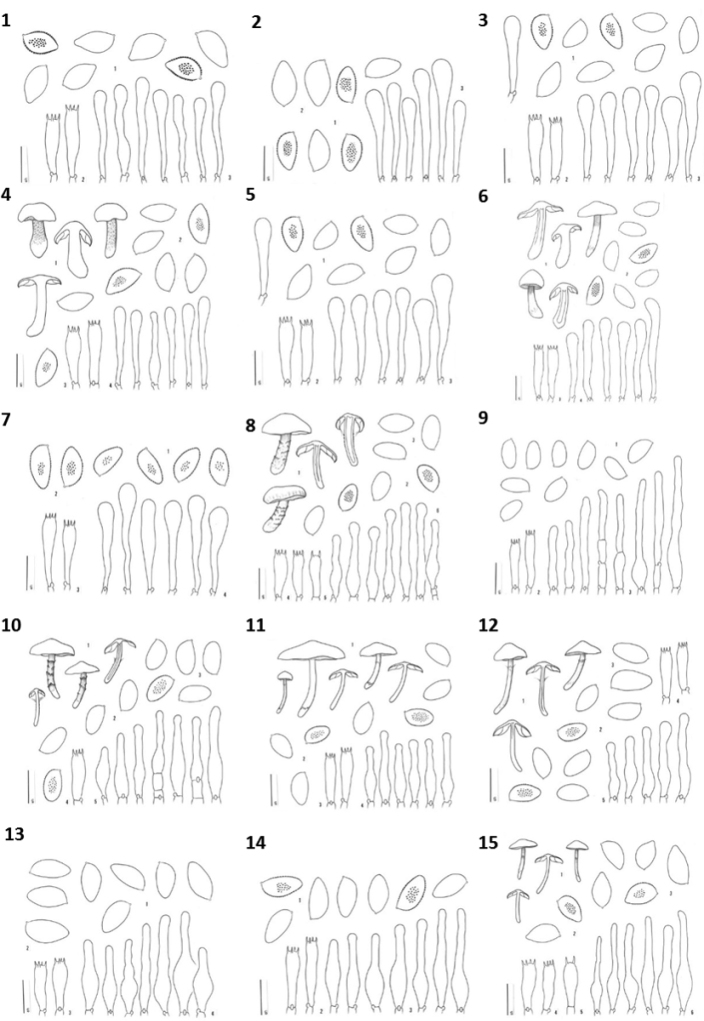
Micro-morphological features (basidiospores, basidia, cheilocystidia) of *Hebeloma* species found in the Rocky Mountain alpine zone. **1***H.vaccinum* (holotype, Herb. PC) **2***H.aurantioumbrinum* ZT12730 **3***H.subconcolor* ZT 13776 **4***H.hiemale* ZT9828 **5***H.avellaneum* DBG-F-019533 **6***H.velutipes* ZT6100 **7***H.alpinum* CLC2875 **8***H.marginatulum* ZT9002 **9***H.alpinicola* ZT13763 **10***H.dunense* ZT9001 **11***H.mesophaeum* ZT8082 **12***H.excedens* ZT7475 **13***H.oreophilum* ZT12733 **14***H.hygrophilum* CLC1462 **15***H.nigellum* ZT6425 **16***H.spetsbergense* micro in Fig. 22. Both two and four-spored basidiospores shown for **2, 5, 7, 8, 10, 12, 13, 15**. Scale bar: 10 µm. All drawings by E. Horak.

##### Rocky Mountain ecology.

In alpine habitats in Colorado, in moss near *Salix* species.

##### Rocky Mountain specimens examined.

U.S.A. COLORADO: San Juan County, San Juan Mountains, Mineral Basin, 31 July 2002, CLC1879 (MONT), C. Cripps. Clear Creek County, Denver Mountain Park, Summit Lake, 3911 m, in *Salixarctica* and *S.glauca*, 20 Aug 2013, DBG-F-027678, L. Gillman.

##### Discussion.

According to Beker et al. (2018), *H.spetsbergense* cannot be distinguished from similar species by ITS. The two RM collections (Fig. 6A) differ by 4 [0] bp, the variation of *H.spetsbergense* within the sample (7 sequences) is 0–5 [0–2] bp. *Hebelomanigellum* is the most similar species occurring in the same habitat and, within this sample, differs in its ITS by 1–8 [0–3] bp from *H.spetsbergense*. Morphologically *H.spetsbergense* is similar to *H.hygrophilum* and *H.nigellum*, but its spores appear to be larger. Previously this species was only known from Svalbard (Beker et al. 2016, 2018), and we report it here from North America for the first time. In Svalbard, it was found with *SalixPolaris* near sea level at a latitude of 78°N. In Colorado, it is reported at elevations of 3700–3800 m and latitudes from 36–38°N, and there is a distance between localities of 6500 km, greatly extending its disjunct range. It remains to be seen, if it also occurs in other arctic and alpine habitats.

With the persistent presence of a cortina and the lageniform or ventricose cheilocystidia, this taxon clearly belongs in H.sect.Hebeloma. The rather strongly dextrinoid amygdaloid spores, less than 14 µm long but more than 7.5 µm wide, distinguish this taxon from the other alpine-arctic species of this section.

## Conclusions

The 16 species of *Hebeloma* we report from the Rocky Mountain alpine zone are from some of the lowest latitudes (latitude 36°–45° N) and highest elevations (3000–4000 m) for arctic-alpine fungi in the northern hemisphere. Twelve of these species have been reported from arctic-alpine habitats in Europe and Greenland, and are now molecularly confirmed from the middle and southern Rockies, greatly expanding their distributions. These are: *Hebelomaalpinum*, *H.aurantioumbrinum*, *H.dunense*, *H.hiemale*, *H.marginatulum*, *H.mesophaeum*, *H.nigellum*, *H.oreophilum*, *H.spetsbergense*, *H.subconcolor*, *H.vaccinum*, and *H.velutipes*. *Hebelomahygrophilum* is known from subalpine habitats in Europe, but has never been recorded in arctic-alpine ecology. Interestingly, hosts can overlap or vary among continents and while Rocky Mountain collections are primarily with *S.arctica*, *S.reticulata*, *S.glauca*, *S.planifolia*, and *Dryasoctopetala*, those from other continents were with these plants or additionally with *S.herbacea*, *S.polaris*, *S.retusa*, *Persicariavivipara*, and *Helianthemum* sp. (Beker et al. 2016; Eberhardt et al. 2015b).

Three species, not known from Europe, have never previously been reported from a true arctic or alpine habitat; they are *H.alpinicola*, *H.avellaneum*, and *H.excedens*. All are species first reported as growing with *Pinaceae* in North America (Peck 1872; Kauffman and Smith 1933; Smith et al. 1983; Hesler unpublished manuscript). We note that the *H.avellaneum* collections described above are from the low alpine and conifers (and conifers are noted in some original descriptions); we do suspect that the ectomycorrhizal association is indeed with *Pinaceae*. The Rockies *H.excedens* collections were all reported with *Salix* in the alpine, yet the holotype was with pine in New York state. This species, like *H.dunense*, *H.mesophaeum*, and *H.nigellum*, appears not to be confined to alpine and arctic habitats. Similarly, *H.alpinicola* appears to be found with a variety of hosts in both alpine and subalpine habitats.

The Rocky Mountain alpine exists as islands on high mountain tops and plateaus far from the arctic and alpine areas of other mountain ranges. While the recent trend, due to molecular analysis, has been to discover differences between European and North American taxa given the same names, in the alpine the reverse appears to be true. Of the ectomycorrhizal genera, a majority of *Inocybe*, *Lactarius*, and *Cortinarius* species from the Rocky Mountain alpine zone have been found to be conspecific with those occurring in arctic and alpine habitats in the European Alps, Pyrenees, Scandinavia, Svalbard, and Greenland through molecular matching of ITS sequences (Cripps et al. 2010; Larsson et al. 2014; Barge et al. 2016; Barge and Cripps 2016). Only a few alpine species of Agaricales and Russulales are so far considered endemic to the Rocky Mountain alpine including *Laccariapseudomontana* Osmundson, C.L. Cripps & G.M. Muell. (Osmundson et al. 2005) and *Lactariuspallidomarginatus* Barge & C.L. Cripps (Barge et al. 2016).

The distributions of various ectomycorrhizal plant hosts in the Rocky Mountains alpine have been shaped by glaciation, topography, parent rock, and climate. Glaciation during the quaternary allowed mixing at the glacial forefronts, interspersed with glacial retreat and withdrawal of cold-adapted plants to mountain tops, which include dwarf *Salix* and *Dryas* (Birks 2008). Tertiary connections have also been suggested (Webber 2003). A view from the North Pole shows Arctic areas as more contiguous than generally considered, and corridors during interglacial periods stretched from the Rockies to the Arctic and Siberia allowing migration and genetic mixing.

Alpine areas, like the arctic, are known to be sensitive to climate change. Greening of these areas is primarily due to shrub encroachment (Tape et al. 2012), and this involves ectomycorrhizal host plants; consequently, ectomycorrhizal fungi communities are likely to change with the loss or gain of different hosts (Geml et al. 2015; Morgado et al. 2015).

## Supplementary Material

XML Treatment for Hebeloma vaccinum

XML Treatment for Hebeloma aurantioumbrinum

XML Treatment for Hebeloma subconcolor

XML Treatment for Hebeloma hiemale

XML Treatment for Hebeloma avellaneum

XML Treatment for Hebeloma velutipes

XML Treatment for Hebeloma alpinum

XML Treatment for Hebeloma marginatulum

XML Treatment for Hebeloma alpinicola

XML Treatment for Hebeloma dunense

XML Treatment for Hebeloma mesophaeum

XML Treatment for Hebeloma excedens

XML Treatment for Hebeloma oreophilum

XML Treatment for Hebeloma hygrophilum

XML Treatment for Hebeloma nigellum

XML Treatment for Hebeloma spetsbergense

## Supplementary materials

Supplementary material 1 GenBank accession numbers of reference sequences of *Hebeloma* spp. from Europe Data type: GenBank accession numbers Explanation note: GenBank accession numbers of reference sequences of *Hebeloma* spp. from Europe (FE dataset). These have been used to assemble species descriptions and distribution patterns of *Hebeloma* sp. in Europe by Beker et al. (2016), where additional information on these collections can be found. The database numbers refer to the project database of H.J. Beker (Beker et al. 2016). This is an open access article distributed under the terms of the Creative Commons Attribution License (CC BY 4.0), which permits unrestricted use, distribution, and reproduction in any medium, provided the original author and source are credited.Cathy L. Cripps, Ursula Eberhardt, Nicole Schütz, Henry J. Beker, Vera S. Evenson, Egon Horak
