# Supplementary material for: The genus Hebeloma in the Rocky Mountain Alpine Zone
Source: MycoKeys. 2019 Feb 11;(46):1–54. doi: 10.3897/mycokeys.46.32823 (PMC6379322; doi:10.3897/mycokeys.46.32823)
Supplement: Supplementary material 1 [file mycokeys-46-001-s001.docx]

**SUPPLEMENTARY DATA**

**Table 3**. Reference sequences of *Hebeloma* spp. from Europe (FE dataset). These have been used to assemble species descriptions and distribution patterns of *Hebeloma* sp. in Europe by Beker et al. (2016), where additional information on these collections can be found. The database numbers refer to the project database of H.J. Beker (Beker et al. 2016).

| Database no. | *Species* | Country | **GenBank acc. no. ITS** |
| --- | --- | --- | --- |
| HJB3733 | *H. aanenii* | Denmark | KM390573 |
| HJB10441 | *H. aanenii* | U.K.: England | KM390539 |
| HJB12164 | *H. aanenii* | Belgium | KM390708, KM390709 |
| HJB12476 | *H. aanenii* | Netherlands | KM390643 |
| HJB12478 | *H. aanenii* | Netherlands | KM390645 |
| HJB12511 | *H. aanenii* | Switzerland | KM390663 |
| HJB12630 | *H. aanenii* | Poland | KM390723 |
| HJB12721 | *H. aanenii* | Czech Republic | KM390741 |
| HJB12729 | *H. aanenii* | France | KM390742 |
| HJB12805 | *H. aanenii* | Netherlands | KM390677 |
| HJB10642 | *H. alpinum* | Switzerland | KM390570, KM390571 |
| HJB11051 | *H. alpinum* | Iceland | JN943865 |
| HJB11085 | *H. alpinum* | Switzerland | KF309411 |
| HJB11087 | *H. alpinum* | Switzerland | KM390599 |
| HJB11088 | *H. alpinum* | Switzerland | KM390597, KM390598 |
| HJB11092 | *H. alpinum* | Switzerland | KM390596 |
| HJB11094 | *H. alpinum* | Switzerland | KM390594, KM390595 |
| HJB11100 | *H. alpinum* | Switzerland | JN943867 |
| HJB11117 | *H. alpinum* | Switzerland | KM390593 |
| HJB11123 | *H. alpinum* | Switzerland | KM390591, KM390592 |
| HJB10906 | *H. aurantioumbrinum* | Norway | KM390563, KM390564 |
| HJB11835 | *H. aurantioumbrinum* | Norway | KM390623 |
| HJB11845 | *H. aurantioumbrinum* | Iceland | KM390627, KM390628 |
| HJB11934 | *H. aurantioumbrinum* | Norway: Svalbard | KM390699 |
| HJB12012 | *H. aurantioumbrinum* | Norway: Svalbard | KM390697 |
| HJB12058 | *H. aurantioumbrinum* | Norway: Svalbard | KM390686, KM390687 |
| HJB12310 | *H. aurantioumbrinum* | Sweden | KM390712 |
| HJB11519 | *H. catalaunicum* | Spain | KX765794 |
| HJB14345 | *H. catalaunicum* | Spain | MK281020 |
| HJB14626 | *H. catalaunicum* | Italy | MK281021 |
| HJB9380 | *H. cavipes* | U.K.: England | KT217360 |
| HJB10453 | *H. cavipes* | U.K.: England | KT217393 |
| HJB11405 | *H. cavipes* | Spain | KT217467 |
| HJB12320 | *H. cavipes* | Denmark | KT217497 |
| HJB12354 | *H. cavipes* | Denmark | KT217503 |
| HJB12358 | *H. cavipes* | Denmark | KT217505 |
| HJB12482 | *H. cavipes* | Netherlands | KT217515 |
| HJB12688 | *H. cavipes* | Poland | KT217524 |
| HJB12800 | *H. cavipes* | Macedonia | KT217526 |
| HJB10814 | *H. clavulipes* | Finland | MK280990 |
| HJB10815 | *H. clavulipes* | Finland | MK280991 |
| HJB10867 | *H. clavulipes* | Sweden | KY271833 |
| HJB11894 | *H. clavulipes* | Sweden | MK281000 |
| HJB13092 | *H. clavulipes* | France | MK281015 |
| HJB13094 | *H. clavulipes* | France | KY271857 |
| HJB13582 | *H. clavulipes* | Germany | MK281016 |
| HJB13587 | *H. clavulipes* | Germany | MK281017 |
| HJB1000028 | *H. clavulipes* | Slovakia | KX765770 |
| HJB1000064 | *H. clavulipes* | France | KX765771 |
| HJB9892 | *H. dunense* | Belgium | MK305907 |
| HJB10922 | *H. dunense* | Belgium | MK305910 |
| HJB10924 | *H. dunense* | Belgium | MK305911 |
| HJB11091 | *H. dunense* | Switzerland | MK305924 |
| HJB11708 | *H. dunense* | Belgium | MK305926 |
| HJB11717 | *H. dunense* | Belgium | MK305925 |
| HJB11778 | *H. dunense* | France | MK305927 |
| HJB13022 | *H. dunense* | France | MK305936 |
| HJB13053 | *H. dunense* | France | MK305937 |
| HJB14141 | *H. dunense* | Belgium | KY271835 |
| HJB9267 | *H. eburneum* | U.K.: England | JN943880 |
| HJB10290 | *H. eburneum* | Belgium | KM390533, KM390534 |
| HJB10525 | *H. eburneum* | France | KF309401 |
| HJB10667 | *H. eburneum* | Sweden | KM390551 |
| HJB10708 | *H. eburneum* | Denmark | KM390555 |
| HJB10840 | *H. eburneum* | Switzerland | KM390559 |
| HJB10899 | *H. eburneum* | France | KM390566 |
| HJB10940 | *H. eburneum* | Denmark | KM390581 |
| HJB10979 | *H. eburneum* | Germany | KM390586 |
| HJB1889 | *H. geminatum* | U.K.: England | KM390524 |
| HJB8633 | *H. geminatum* | Belgium | KM390526 |
| HJB10603 | *H. geminatum* | Belgium | KM390770 |
| HJB10766 | *H. geminatum* | U.K.: England | KM390554 |
| HJB10833 | *H. geminatum* | Denmark | KF309405 |
| HJB11736 | *H. geminatum* | Belgium | KM390616, KM390617 |
| HJB11829 | *H. geminatum* | Norway | KM390750 |
| HJB12469 | *H. geminatum* | Netherlands | KM390640 |
| HJB12934 | *H. geminatum* | France | KM390746 |
| HJB13309 | *H. geminatum* | U.K.: England | KM390684 |
| HJB13114 | *H. grandisporum* | Romania | KT071023 |
| HJB8816 | *H. helodes* | Belgium | KM390529 |
| HJB10606 | *H. helodes* | Belgium | KM390542 |
| HJB10680 | *H. helodes* | U.K.: England | KM390548 |
| HJB10725 | *H. helodes* | Norway | KM390568 |
| HJB10726 | *H. helodes* | Denmark: Faroe Islands | KM390772 |
| HJB10727 | *H. helodes* | Finland | KM390569 |
| HJB10731 | *H. helodes* | U.K.: England | KM390556 |
| HJB11906 | *H. helodes* | U.K.: Scotland | KM390630 |
| HJB12520 | *H. helodes* | France | KM390666 |
| HJB12521 | *H. helodes* | Netherlands | KM390667 |
| HJB12524 | *H. helodes* | Netherlands | KM390670 |
| HJB12525 | *H. helodes* | Netherlands | KM390671 |
| HJB12539 | *H. helodes* | Netherlands | KM390674 |
| HJB12905 | *H. helodes* | Belgium | KM390614 |
| HJB13151 | *H. helodes* | Poland | KM390763 |
| HJB8455 | *H. hiemale* | U.K.: England | GQ869512, GQ869513 |
| HJB8890 | *H. hiemale* | Belgium | GQ869484 |
| HJB8920 | *H. hiemale* | Belgium | GQ869487, GQ869488 |
| HJB9384 | *H. hiemale* | U.K.: England | GQ869482 |
| HJB9479 | *H. hiemale* | Spain | KT217363 |
| HJB10296 | *H. hiemale* | Belgium | KT217384 |
| HJB10370 | *H. hiemale* | Belgium | EU570168 |
| HJB10440 | *H. hiemale* | U.K.: England | KT217390 |
| HJB10472 | *H. hiemale* | U.K.: England | EU570169 |
| HJB10764 | *H. hiemale* | Norway | GQ869491 |
| HJB10893 | *H. hiemale* | Denmark | KT217442 |
| HJB11180 | *H. hiemale* | U.K.: Scotland | GQ869497 |
| HJB11236 | *H. hiemale* | Belgium | GQ869495, GQ869496 |
| HJB11499 | *H. hiemale* | Belgium | GQ869500 |
| HJB11606 | *H. hiemale* | U.K.: England | GQ869501, GQ869502 |
| HJB11695 | *H. hiemale* | Belgium | GQ869503 |
| HJB11704 | *H. hiemale* | France | GQ869505 |
| HJB11905 | *H. hiemale* | U.K.: Scotland | GQ869514 |
| HJB11948 | *H. hiemale* | Norway: Svalbard | GQ869519 |
| HJB11965 | *H. hiemale* | Norway: Svalbard | GQ869520 |
| HJB12370 | *H. hiemale* | Finland | GQ869533 |
| HJB13821 | *H. hiemale* | Germany | KT217561 |
| HJB9325 | *H. hygrophilum* | U.K.: England | MK281022 |
| HJB9326 | *H. hygrophilum* | U.K.: England | MK281023 |
| HJB10932 | *H. hygrophilum* | Finland | MK280993 |
| HJB11209 | *H. hygrophilum* | Finland | MK280997 |
| HJB11891 | *H. hygrophilum* | Sweden | MK280999 |
| HJB12347 | *H. hygrophilum* | Estonia | MK281007 |
| HJB12964 | *H. hygrophilum* | France | MK281013 |
| HJB12965 | *H. hygrophilum* | France | KY271855 |
| HJB12966 | *H. hygrophilum* | France | KX765778 |
| HJB12967 | *H. hygrophilum* | France | MK281014 |
| HJB10132 | *H. incarnatulum* | Finland | KT218239 |
| HJB10133 | *H. incarnatulum* | Finland | KT218240 |
| HJB10136 | *H. incarnatulum* | Finland | KT218243 |
| HJB10748 | *H. incarnatulum* | Estonia | KT218310 |
| HJB10788 | *H. incarnatulum* | Denmark | KT218315 |
| HJB10789 | *H. incarnatulum* | Denmark | KT218316 |
| HJB12907 | *H. incarnatulum* | Switzerland | KT218417 |
| HJB13589 | *H. incarnatulum* | Germany | KT218441 |
| HJB13629 | *H. incarnatulum* | Germany | KT218448 |
| HJB11034 | *H. islandicum* | Iceland | KX765799 |
| HJB9289 | *H. leucosarx* | U.K.: England | KT218219 |
| HJB9378 | *H. leucosarx* | U.K.: England | KT218226 |
| HJB10140 | *H. leucosarx* | Finland | KT218247 |
| HJB10155 | *H. leucosarx* | Belgium | KT218249 |
| HJB10750 | *H. leucosarx* | U.K.: Scotland | KT218311 |
| HJB11160 | *H. leucosarx* | U.K.: Scotland | KT218334 |
| HJB11309 | *H. leucosarx* | Belgium | KT218341 |
| HJB11566 | *H. leucosarx* | France | KT218355 |
| HJB11626 | *H. leucosarx* | Belgium | KT218358 |
| HJB12076 | *H. leucosarx* | France | KT218383 |
| HJB10937 | *H. marginatulum* | Finland | MK305912 |
| HJB10967 | *H. marginatulum* | Iceland | MK305913 |
| HJB11143 | *H. marginatulum* | Switzerland | MK305919 |
| HJB11144 | *H. marginatulum* | Switzerland | KY271834 |
| HJB11145 | *H. marginatulum* | Switzerland | MK305918 |
| HJB11146 | *H. marginatulum* | Switzerland | MK305917 |
| HJB11147 | *H. marginatulum* | Switzerland | MK305916 |
| HJB11149 | *H. marginatulum* | Switzerland | MK305915 |
| HJB11150 | *H. marginatulum* | Switzerland | MK305914 |
| HJB11151 | *H. marginatulum* | Switzerland | KT071029 |
| HJB11938 | *H. marginatulum* | Norway: Svalbard | MK305933 |
| HJB11946 | *H. marginatulum* | Norway: Svalbard | MK305934 |
| HJB11954 | *H. marginatulum* | Norway: Svalbard | MK305935 |
| HJB12009 | *H. marginatulum* | Norway: Svalbard | KT071030 |
| HJB12010 | *H. marginatulum* | Norway: Svalbard | MK305929 |
| HJB12011 | *H. marginatulum* | Norway: Svalbard | MK305930 |
| HJB12014 | *H. marginatulum* | Norway: Svalbard | MK305931 |
| HJB12015 | *H. marginatulum* | Norway: Svalbard | MK305928 |
| HJB12961 | *H. marginatulum* | France | KY271854 |
| HJB13106 | *H. marginatulum* | Italy | MK305938 |
| HJB13119 | *H. marginatulum* | Poland | KT071028 |
| HJB9403 | *H. mesophaeum* | Spain | MK305906 |
| HJB10697 | *H. mesophaeum* | U.K.: England | MK305908 |
| HJB10705 | *H. mesophaeum* | U.K.: England | MK305909 |
| HJB10854 | *H. mesophaeum* | Denmark | EF451057 |
| HJB11121 | *H. mesophaeum* | Switzerland | MK305923 |
| HJB11130 | *H. mesophaeum* | Switzerland | MK305922 |
| HJB11138 | *H. mesophaeum* | Switzerland | MK305920 |
| HJB13732 | *H. mesophaeum* | U.K.: England | MK305939 |
| HJB14243 | *H. mesophaeum* | Romania | KT071038 |
| HJB10865 | *H. minus* | Iceland | JN943872 |
| HJB10866 | *H. minus* | Iceland | KM390753 |
| HJB11079 | *H. minus* | Iceland | JN943866 |
| HJB11107 | *H. minus* | Switzerland | JN943868 |
| HJB11945 | *H. minus* | Norway: Svalbard | KM390700 |
| HJB12007 | *H. minus* | Norway: Svalbard | JN943857 |
| HJB12512 | *H. minus* | France | KM390664 |
| HJB12568 | *H. minus* | Switzerland | KM390771 |
| HJB13577 | *H. minus* | Norway: Svalbard | KM390765 |
| HJB1000065 | *H. minus* | France | KM390751 |
| HJB11153 | *H. nanum* | U.K.: Scotland | MK280996 |
| HJB13671 | *H. nanum* | Italy | MK281018 |
| HJB1000155 | *H. nanum* | Czech Republic | KX765798 |
| HJB13807 | *H. naviculosporum* | Germany | KT071039 |
| HJB14211 | *H. naviculosporum* | Spain | KT071040 |
| HJB14568 | *H. naviculosporum* | Slovakia | KT071041 |
| HJB1000023 | *H. naviculosporum* | Spain | KX765797 |
| HJB9516 | *H. nigellum* | U.K.: England | MK281024 |
| HJB10805 | *H. nigellum* | Denmark | KT071043 |
| HJB10851 | *H. nigellum* | Iceland | MK280992 |
| HJB10936 | *H. nigellum* | Finland | MK280994 |
| HJB10944 | *H. nigellum* | Finland | KT071044 |
| HJB10960 | *H. nigellum* | Switzerland | MK280995 |
| HJB12353 | *H. nigellum* | Sweden | MK281008 |
| HJB12488 | *H. nigellum* | France | MK281010 |
| HJB12827 | *H. nigellum* | U.K.: Scotland | MK281011 |
| HJB12914 | *H. nigellum* | Switzerland | MK281012 |
| HJB1000042 | *H. nigellum* | France | KX765786 |
| HJB11831 | *H. oreophilum* | Finland | MK280998 |
| HJB11950 | *H. oreophilum* | Norway: Svalbard | MK281001 |
| HJB11952 | *H. oreophilum* | Norway: Svalbard | KY271844 |
| HJB11953 | *H. oreophilum* | Norway: Svalbard | KY271845 |
| HJB11976 | *H. oreophilum* | Norway: Svalbard | KY271850 |
| HJB11977 | *H. oreophilum* | Norway: Svalbard | MK281003 |
| HJB12400 | *H. oreophilum* | Finland | MK281009 |
| HJB13117 | *H. oreophilum* | Slovakia | KT071045 |
| HJB13126 | *H. oreophilum* | Slovakia | KT071046 |
| HJB14234 | *H. oreophilum* | Slovakia | KT071047 |
| HJB11992 | *H. pallidolabiatum* | Norway: Svalbard | KM390702, KM390703 |
| HJB12059 | *H. pallidolabiatum* | Norway: Svalbard | KM390713 |
| HJB1000071 | *H. perpallidum* | Austria | KM390620, KM390621 |
| HJB11932 | *H. pubescens* | Norway: Svalbard | KY271841 |
| HJB11933 | *H. pubescens* | Norway: Svalbard | MK305932 |
| HJB12008 | *H. pubescens* | Norway: Svalbard | KX765792 |
| HJB11973 | *H. spetsbergense* | Norway: Svalbard | MK281002 |
| HJB11979 | *H. spetsbergense* | Norway: Svalbard | MK281004 |
| HJB11980 | *H. spetsbergense* | Norway: Svalbard | MK281005 |
| HJB11981 | *H. spetsbergense* | Norway: Svalbard | MK281006 |
| HJB11982 | *H. spetsbergense* | Norway: Svalbard | KX765793 |
| HJB10714 | *H. subconcolor* | Denmark: Faroe Islands | KT218308 |
| HJB11114 | *H. subconcolor* | Switzerland | KT218330 |
| HJB11141 | *H. subconcolor* | Switzerland | KT218331 |
| HJB11142 | *H. subconcolor* | Switzerland | KT218332 |
| HJB12293 | *H. subconcolor* | Finland | KT218386 |
| HJB12413 | *H. subconcolor* | Greenland | KT218391 |
| HJB12565 | *H. subconcolor* | Switzerland | KT218405 |
| HJB1000044 | *H. subconcolor* | France | KT218473 |
| HJB5115 | *H. vaccinum* | U.K.: Wales | KT071052 |
| HJB10256 | *H. vaccinum* | Belgium | KT217380 |
| HJB10589 | *H. vaccinum* | U.K.: Wales | KT071050 |
| HJB10593 | *H. vaccinum* | U.K.: Wales | KT217410 |
| HJB10713 | *H. vaccinum* | Denmark | KT217424 |
| HJB11135† | *H. vaccinum* | Switzerland | MK305921 |
| HJB11318 | *H. vaccinum* | Belgium | KT217462 |
| HJB11578 | *H. vaccinum* | Belgium | MF039237 |
| HJB12348 | *H. vaccinum* | Denmark | KT217500 |
| HJB1000247 | *H. vaccinum* | Germany | KT217576 |
| HJB1000132 | *H. vejlense* | Denmark | EU881922 |
| HJB9294 | *H. velutipes* | U.K.: England | KT218222 |
| HJB9926 | *H. velutipes* | U.K.: England | KT218235 |
| HJB10126 | *H. velutipes* | Belgium | KT218237 |
| HJB10129 | *H. velutipes* | Belgium | KT218238 |
| HJB10273 | *H. velutipes* | Belgium | KT218255 |
| HJB10291 | *H. velutipes* | Belgium | KT218259 |
| HJB10350 | *H. velutipes* | Belgium | KT218274 |
| HJB10485 | *H. velutipes* | U.K.: England | KT218288 |
| HJB10588 | *H. velutipes* | U.K.: Wales | KT218294 |
| HJB11022 | *H. velutipes* | Iceland | KT218327 |
| HJB11157 | *H. velutipes* | U.K.: Scotland | KT218333 |
| HJB11339 | *H. velutipes* | Spain | KT218344 |
| HJB11951 | *H. velutipes* | Norway: Svalbard | KT218376 |
| HJB11960 | *H. velutipes* | Norway: Svalbard | KT218377 |
| HJB12071 | *H. velutipes* | France | KT218381 |
| HJB12526 | *H. velutipes* | Germany | KT218399 |
| HJB12527 | *H. velutipes* | Germany | KT218400 |
| HJB13122 | *H. velutipes* | Slovakia | KT071053 |
| HJB13874 | *H. velutipes* | Poland | KT218457 |
| HJB14206 | *H. velutipes* | Cyprus | KT218466 |

†This collection is only used as photo reference.

**Spore measurements for individual collections by E. Horak.**

Spore measurements for 4-spored basidia only. Data strictly distinguish between basidiospores born on 4-spored basidia and 2-spored basidia [in the “statistiques” the reported measurements represent a mixture of both and accordingly the wide range of referred data in the text

1. *H. vaccinum* (ZT 13764): Spores 12-13 x 6-7 µm. Cheilocystidia (30-)38-65 x 6-8 µm (at apex).

2. *H. aurantioumbrinum* (ZT 12730): Spores 11-12 x 5.5-6(-6.5) µm. Cheilocystidia 45-60 x 7-8.4 µm (at apex).

3. *H. subconcolor* (ZT 13776): Spores 9-11.5 x 5.5-6 µm. Cheilocystidia 45-65 x 8-10 µm (-12 µm, at apex.

4. *H. hiemale* (ZT 9828): Spores 10.5-12 x 5.5-6 µm. Cheilocystidia (40-)45-65 x (6-)7-9 µm (at apex).

5. *H. avellaneum* (DBG-F-019533): Sp. 7.5-9 x 4-4.5 µm. Cheilocystidia (30-)35-60(-65) x 7-10 µm (at apex).

6. *H. velutipes* (ZT 6100): Spores 10-12 x 5.5-6 µm. Cheilocystidia (35-)50-70(-80) x 8-12 µm (at apex).

7. *H. alpinum* cf. (CLC 2855): Spores10-11 x 5-5.5 µm. Cheilocystidia 45-65 x 8-11 µm (at apex).

8. *H. marginatulum* (ZT 9002) Spores 9-11.5 x 5-6 µm (at apex). Cheilocystidia 35-60 x 5-7.5 (at apex) .

9. *H. nigromaculatum* (ZT 13763): Sp. 8-9(-9.5) x 4.5-5 µm. Cheilocystsidia 40-80 x 4.5-6 µm (-8 µm, at base).

10. *H. dunense* (ZT 9001): Spores 9-10.5 x 5-5.5 µm. Cheilocystsidia (25-)30-55 x 4-6(-7) µm.

11. *H. mesophaeum* (ZT 8082): Spores (8.5-)9-10 x 5-5.5 µm. Cheilocystidia 32-50 x 5-6 µm (median -8 µm).

12. *H. excedens* (ZT 7475): Spores 8.5-10 x (4-)4.5-5.5 µm. Cheilocystidia 32-55(-60) x 5-8 µm (median).

13. *H. oreophilum* (ZT 12733): Spores 10.5-12 x 5.5-6.5 µm. Cheilocystidia (35-)45-65 x 7-10 µm (median -16 µm).

14. *H. hygrophilum* (CLC1462): Spores 10-12.5 x 5-6 µµ. Cheilocystidia 35-50 x 7.5-10 µm (median).

15. *H. nigellum* (ZT 6425): Spores 9.5-11 x 5.5-7 µm. Cheilocystidia 35-65 x 7-10 µm (median).
